# The Microbial Anti-Inflammatory Molecule (MAM) is a key protein processed and exported to *Faecalibacterium duncaniae* envelope

**DOI:** 10.1080/19490976.2025.2519695

**Published:** 2025-06-18

**Authors:** Thaís Vilela Rodrigues, Sylvain Marthey, Sandrine Auger, Luis Lima de Jesus, Céline Henry, Christine Pechoux, Ana Arteni, Véronique Martin, Philippe Langella, Siomar de Castro Soares, Gwenaëlle Andre, Vasco Ariston de Carvalho Azevedo, Jean-Marc Chatel

**Affiliations:** a Université Paris Saclay, INRAE, AgroParisTech, MICALIS, Jouy-enJosas, France; b Instituto de Ciências Biológicas, Federal University of Minas Gerais, Belo Horizonte, Brazil; c Alimentation humaine, Université Paris Saclay, INRAE, MAIAGE, Jouy-en-Josas, France; d Alimentation humaine, Université Paris Saclay, INRAE, GABI, Jouy-en-Josas, France; e Université Paris-Saclay, CEA, CNRS, Institute for Integrative Biology of the Cell, Gif-sur-Yvette, France; f Instituto de Ciências Biológicas e Naturais, Federal University of Triângulo Mineiro, Uberaba, Brazil

**Keywords:** Cell envelope, proteomics, ABC transporter, leader and cargo peptides, *Faecalibacterium*, microbiota, inflammation

## Abstract

MAM (Microbial-Anti-Inflammatory Molecule) is a key effector protein with anti-inflammatory properties in *Faecalibacterium duncaniae*, a critical human gut microbiota species. Despite its importance, MAM function and molecular features remain poorly understood. This study elucidates MAM’s physiological importance by examining its cellular localization, secretion dynamics, and structural organization. Mass spectrometry and immunogold labeling confirmed MAM as the most abundant protein in the cell envelope and the second most abundant in the overall proteome, localizing it to the bacterial surface. Bioinformatic and *in silico* analyses suggest that MAM contains an N-terminal leader peptide with motifs recognized by a Peptidase-domain-Containing ABC Transporter (PCAT), enabling cargo transport to the cell envelope. After N-terminal excision, the cargo protein could be transported to the cell envelope via this PCAT, where it could assemble into a hexameric structure, as revealed by docking and AlphaFold3 modeling. Such results were supported by electron microscopy showing a lattice-like organization on the bacterial surface. This work introduces a novel discussion about the singular organization of the *F. duncaniae* cell envelope, having MAM as a key component for the bacteria, supporting the understanding of the unique biology of *F. duncaniae* and its potential as a next-generation probiotic or live biotherapeutic.

## Introduction

*Faecalibacterium duncaniae*, formerly known as *Faecalibacterium prausnitzii*, is an extremely oxygen-sensitive (EOS), non-motile, and non-spore-forming bacterium that belongs to the *Clostridium* cluster IV within the *Bacillota* (*Firmicutes*) phylum. As a commensal organism, *F. duncaniae* is a major component of the healthy human gut, able to represent up to 5% of the total bacterial microbiota.^1^ The species has garnered attention as a next-generation probiotic or live biotherapeutics, due to its pivotal role in maintaining gut health and modulating inflammation, in a colitis murine model, mimicking symptoms of Inflammatory Bowel Diseases (IBDs) such as Crohn’s disease (CD) and Ulcerative Colitis (UC).^2^ Additionally, a lower abundance of *F. duncaniae* has been positively associated with various conditions, such as obesity, diabetes, Parkinson’s disease, and more recently, COVID-19 and influenza virus infections.^3–7^

Different beneficial mechanisms related to *F. duncaniae* toward the host health have been described. The bacterium is a substantial producer of butyrate,^1,8^ a key short-chain fatty acid (SCFA) that serves as the primary energy source for colonocytes, protects the intestinal mucosa by enhancing mucin production, and plays an anti-inflammatory role by inhibiting the NF-kB pathway.^9,10^ Another crucial factor is the production of bioactive peptides attributed to MAM (Microbial Anti-Inflammatory Molecule). MAM is a 14.5 kDa protein produced exclusively by *Faecalibacterium* species. MAM has demonstrated significant anti-inflammatory properties, particularly through the inhibition of the NF-κB pathway.^11,12^ However, detailed insights into its physiological role within bacteria, including its structure, function, potential processing, and cognate protein–protein partnering mode, remain to be deciphered.

MAM was primarily identified by its peptides in the *F. duncaniae* supernatant.^12^ Early *in silico* predictions revealed that MAM structure consists primarily of hydrophobic residues, with a tertiary structure indicating a globular conformation, having a β-sheet central core flanked by α-helices.^12,13^ Although the physiological role of MAM in the species remains unknown, the encoding gene for MAM was observed to be highly expressed during both exponential and stationary growth phases, even in a poor nutritional environment.^14^ This suggests that MAM likely plays an important role in the physiology of the bacteria. The genomic region of the MAM coding sequence includes genes associated with various functions. In most *Faecalibacterium* species, the gene encoding a PCAT is located adjacent to the MAM gene.^11^ PCATs are key players in a specific transport system where a core ABC exporter is covalently linked to a C39 peptidase and an ATP interaction chain (nucleotide-binding domains – NBD). The C39 domain acts as cysteine protease, which recognizes and cleaves a leader peptide, specifically after a double G motif, to facilitate the cargo protein transport.^15^ Although a potential interaction between MAM and PCAT has been suggested, and a cargo peptide identified at the N-terminal end of MAM,^11^ this relationship remains unclarified.

MAM exhibits significant diversity among the different species of *Faecalibacterium*. Phylogenetic and comparative analyses of MAM demonstrated the existence of 10 different clusters of MAM sequences within the genus. This diversity is observed in the molecular composition and in the anti-inflammatory activity of MAM, for which different inhibition levels of the NF-kB pathway were observed among *Faecalibacterium* species.^11^

In previous studies, MAM peptides demonstrated significant anti-inflammatory properties in various colitis murine models and in *in vitro* assays.^11,16,17^ The activity of MAM was primarily tested by plasmid transfection encoding the entire protein in epithelial cell lines.^12^ These studies revealed MAM’s capacity to inhibit the NF-κB pathway in a dose-dependent manner. This influence on the NF-κB pathway was also demonstrated *in vivo* through oral administration of *Lactococcus lactis*, carrying a plasmid containing the cDNA of MAM. Under the control of an eukaryotic promoter, it enables the production of MAM directly by the host.^16^ Additionally, a luciferase reporter assay targeting the NF-κB pathway was also performed, demonstrating MAM anti-inflammatory activity in DNBS (Dinitrobenzene sulfonic acid) and DSS (Dextran Sodium Sulfate)-induced colitis models.^11,16^

MAM has also been correlated with the maintenance of the gut barrier integrity. Studies, using the gut microbiota of a diabetes mellitus mouse model and intestinal cell lines, showed a positive correlation between barrier stability and permeability through the interaction of recombinant MAM with tight junction proteins, demonstrating MAM’s multifaceted role in maintaining intestinal integrity.^18^

While MAM’s biotherapeutic potential in gut health is evident, its functional contribution to *F. duncaniae* physiology remains unexplored. To address this issue, we employed various biochemical and molecular cell biology approaches in this work to investigate MAM’s functional and structural aspects. We performed cell fractionation, proteomic analysis, and immunogold labeling with polyclonal antibodies to evaluate the localization and abundance of MAM within the cell. We also led structural bioinformatics studies to explore the possibility of MAM being recognized and exported outside the cytoplasm by PCAT, and we investigated MAM’s capacity to fold and eventually self-assemble into a supramolecular organization. We revealed intriguing aspects of the envelope of *F. duncaniae* that do not follow the classical bacterial cell envelope organization. Besides, the main proteinaceous component of the cell surface is MAM, which forms an ordered lattice on the surface of *F. duncaniae*, suggesting a possible role in maintaining envelope integrity. Thus, this study highlights the need for further investigation into the architecture of the *F. duncaniae* cell envelope, opening new discussions about this unique bacterium.

## Methods

### Bacterial growth and culturing conditions

*F. duncaniae* A2–165 (DSM #17677) was cultivated on a BHIS agar plate enriched with (Brain Heart Infusion broth 37 g/L, Difco) supplemented with yeast extract (5 g/L, Difco) and 3% GAC (Glucose 6.6%, Acetate 5.5%, and cysteine 1.66%). BHIS is a rich, complex medium that supports the growth of fastidious anaerobes.^19^ The addition of glucose and acetate provides essential carbon sources, while cysteine contributes to the reducing environment required by this extremely oxygen-sensitive bacterium. This combination has been studied and previously observed in the cultivation of *F. duncaniae* in previous studies.^2,20,21^ The anaerobic chamber was set with N_2_ (90%), CO_2_ (5%), and H2 (5%).^22^ Single colonies were inoculated in a liquid medium (BHIS-GAC) at 37°C. Overnight pre-cultures were used to prepare 10% cultures with an initial optical density (OD_600 nm_) of approximately 0.1. The experiments were conducted with four replicates.

### Supernatant sample collection and preparation for mass spectrometry analysis

For each of the four replicates, 10 ml of culture was collected. At the exponential phase (EX), samples were collected at 6 and 9 h after inoculation, and at the stationary phase, the collection was made at 18 (Early Stationary – ES) and 25 h (Late Stationary – LS). Cultures were harvested for 15 min at 5000 ×g at 4°C, and the supernatant was collected. The Oasis Cartridge Prime HLB column was previously wetted with acetonitrile (ACN) and then equilibrated with 2% ACN and 0.1% formic acid. Four hundred and fifty microliters of supernatant was loaded and eluted sequentially with 200 µl of 40% ACN, followed by 200 µl of 80% ACN. Eluted samples were submitted to a 10 kDa Millipore cutoff centrifugation at 12,000 rpm for 40 min at 4°C to remove any remaining protein. The filtrate was dried using a speed-vac, followed by the addition of 200 µL of loading buffer (2% ACN and 0.08% trifluoroacetic acid). The filtrate was then diluted 20-fold with loading buffer (2% ACN and 0.08% trifluoroacetic acid (TFA), and 4 µL were injected into the liquid chromatography-tandem mass spectrometry (LC-MS/MS).

Supernatant peptides were identified using an Orbitrap Fusion Lumos Tribrid mass spectrometer (Thermo Fisher Scientific, San Jose, CA) coupled to an UltiMate3000 RSLCnano ultraHPLC system (Dionex, Sunnyvale, CA). Each 4 μL sample was injected for online desalting onto a PepMap C-18 reverse-phase (RP) nanotrap column (3 μm, 75 μm × 20 mm, Dionex) with nanoViper fittings (flow rate: 20 μL min^−1^), separated on a PepMap C-18 reverse-phase nano column (3 μm, 75 μm × 50 cm), and eluted with a 50 min gradient of 5–35% ACN in 0.1% formic acid at 250 nL min^−1^, a 5-min ramp to 40% ACN/0.1% formic acid, a 3-min ramp to 98% ACN/0.1% formic acid, and a 5-min hold at 98% AC/0.1% formic acid. The mass spectrometer was operated in positive ion mode, with the nanospray voltage set to 1.6 kV and the source temperature set to 270°C. The instrument was operated in data-dependent acquisition mode and high-energy collisional dissociation fragmentation mode (collision energy: 30%). In all experiments, full MS scans were acquired over the 400–2000 *m/z* mass range, with detection in the Orbitrap mass analyzer at a resolution of 120,000, an automatic gain control set to 1 × 10^5^ and an intensity threshold of 20,000. Each precursor ion scan was followed by a 2.5 s “top speed” data-dependent Orbitrap MS/MS run, with a 1.6 *m/z* window for the quadrupole isolation of precursor peptides with multiply charged ions from 1 to 3. Fragment ion spectra were acquired in the Orbitrap mass analyzer at a resolution setting of 30,000, an automatic gain control set to 5 × 10,^4^ and a dynamic maximum injection time. Polysiloxane ions *m/z* 445.12002, 519.13882, and 593.15761 were used for internal calibration.

The data were converted into mzXML format using MS Convert (ProteoWizard, version 3.0.8934). Proteins were identified using X!Tandem v.2017.2.1.4^23^ by matching peptides against the MAM database. Proteins were filtered and grouped using open-source X!TandemPipeline software (version 0.4.62, http://pappso.inrae.fr/bioinfo/xtandempipeline/).^24^ The data were compared with a contaminant database to eliminate spectra due to contaminants.

The peptide identification process was run with a precursor mass tolerance of 10 ppm and a fragment mass tolerance of 10 ppm. No digestion rule was applied. The fix modification was set to cysteine carbamidomethylation, and methionine oxidation was considered a potential modification. In a second pass, N-terminal acetylation was added as another potential modification, whereas all the other above-mentioned settings remained unchanged. Identified proteins were filtered as follows: (i) peptide E-value <0.01 with a minimum of two peptides per protein, and (ii) a protein E-value of 10^−4^.

### Data normalization

To enable the direct comparison between OD and peptide abundance over time, all values (OD, mean peptide count, and standard deviation) were normalized to their respective maximum. This allowed the visualization of both datasets on the same relative scale.

### Envelope and cytoplasmic protein extraction and preparation for mass spectrometry analysis

Here, the same culture procedures were conducted as previously described. After the harvesting of 30 ml of culture at the early stationary phase (18 h of growth), the cell pellet was washed twice with phosphate-buffered saline (PBS) and then resuspended in lysis buffer (Tris-HCl 50 mm pH 8.0; 50 μl/ml Protease Inhibitor Cocktail). To break the cells, three cycles of 10 s of sonication were performed at a frequency of 20 kHz. The supernatant containing cytoplasmic proteins was recovered by centrifugation at 20,000 ×g for 15 min. Pellets containing the envelope fraction were washed twice with PBS and then resuspended in PBS. Quantification was performed in triplicate using UV absorbance at 280 nm. After quantification, 10 μg of protein was submitted to a short migration electrophoresis using a 1D gel (NuPAGE® 4–12% Bis-Tris Gel, Novex), with Coomassie G-250 (SimplyBlueTM SafeStain, Invitrogen) used as a dye. The gel was then cut into small pieces and destained with Solvent A (10% v/v acetic acid, 40% v/v ethanol) and Solvent B (50% v/v 50 mm ammonium bicarbonate, 50% v/v ACN). Next, 10 mm dithiothreitol and 55 mm iodoacetamide (Sigma) were used for reduction and alkylation, followed by overnight digestion with 100 ng trypsin (Promega). Peptides were extracted with 0.5% v/v trifluoroacetic acid and 50% v/v ACN. The samples were dried using a dry-vacuum system (SavantTM SPD121D, Thermo Fisher Scientific), and peptides were resuspended in 50ul of loading buffer (0.08% v/v trifluoroacetic acid, 2% v/v ACN) for LC-MS/MS injection. Protein identification followed the process described in the previous section.

*In-silico* analysis of MAM motifs and modeling as monomeric and oligomeric structures

3D modeling of MAM was performed with AlphaFold 3 (AF3). The fasta sequence of MAM from *F. duncaniae* was retrieved from UniProt under the ID C7H4X2, and submitted to the AF3 online server (https://alphafoldserver.com/.)^25,26^ to be modeled, first as a monomer with the complete sequence (1–135 aa) then as homopolymers composed of 2 to 8 MAM sequences (22–135 aa). For the predictions of these complexes, the MAM sequence was trimmed off the first 21 amino acids corresponding to the putative signal peptide. Similarly, the transporter PCAT (ID C7H4X1) was retrieved from UniProt^25^ and the modeling of the PCAT dimer bound to the MAM monomer was subjected to the AF3 server.^26^ For each prediction, five structures were computed and ranked for each model, according to the global complex ranking metric with chiral mismatch and steric clash penalties generated by AF3. The best model ranked by AF3 was retained for subsequent analyses. Visual analyses were performed using PyMOL.^27^ Structural homologs that could have been deposited on the protein data bank were searched for using the Foldseek server (https://search.foldseek.com/).^28^ To evaluate the predicted oligomeric structures, PDBePISA (Proteins, Interfaces, Structures, and Assemblies) was used to assess the interactions between the complexes’ units.^29^ ProtParam was utilized to compute the theoretical pI and the number of charged residues.^30^

### Interaction of MAM leader peptide with the PCAT

Initially, MAM sequence was screened for signature motifs associated with the leader peptide ([MMMPANx(8,11)VxGG]) and PCAT ([GIE[T/L][V/I]K]) using the Fuzzpro tool.^31^ For the docking, the best predicted model of MAM bound to the PCAT transporter was superimposed onto the cryo-EM structure of its structural homolog PCAT1, bound to CtA peptide substrate in *Clostridium thermocellum*.^32^ This was performed using the PyMOL align command. Next, the MAM signal peptide of 21 residues-long was cut and kept to measure and characterize its molecular interaction with PCAT. To study it at the excision site, the peptide encompasses on-purpose the extra Ala22, located right after the cleavage site at the double G20G21. The binding energy between the MAM peptide and PCAT was evaluated using Autogrid4 with the largest cubic grid, centered on the middle of the peptide considered rigid, and Autodock4 with the function epdb.^33^ To get a positive control, the same protocol was used to measure the interaction between PCAT1 (chain C) and CtA (chain D between Asn8 and Thr26) of the experimental complex (PDB ID: 6v9z) from *Acetivibrio thermocellus* ATCC27405.^34^ The cryo-EM and AF3 complexes were visually inspected using PyMOL.

Lastly, to evaluate the importance of the double glycine motif (G20G21) in the interaction with PCAT, we performed *in silico* mutations in the MAM leader peptide. These included deletion of the G20G21 motif (ΔGG) and residue substitutions (GG→AA and GG→EE). The mutated sequences were submitted to AlphaFold3 for interaction prediction. PyMol was used for residue distance measurements.

### Lithium chloride (LiCl) extraction and protein identification

This chaotropic agent LiCl was chosen to investigate the envelope of *F. duncaniae* more deeply. Overnight cultures of *F. duncaniae* at stationary phase (500 mL) were harvested by centrifugation at 5000 × g for 15 min at 4°C. The supernatant was discarded, and two wash cycles with 50 mL of PBS at pH 7.4 were conducted. The remaining pellet was resuspended in 5 M LiCl and agitated for 15 min at 4°C. To obtain the enriched envelope extraction, the supernatant was collected after harvesting at 9000 ×g for 15 min (4°C). Supernatants were ultracentrifuged at 49,000 × g for 3 h to concentrate the envelope proteins. The resulting pellet was resuspended in PBS, and protein concentration was determined by measuring absorbance at 280 nm using a NanoDrop spectrophotometer (Thermo Fisher Scientific, Waltham, MA, USA). For SDS-PAGE analysis, 10 µg of protein was loaded onto a pre-cast 4–15% Mini-PROTEAN^Ⓡ^ TGX™ gel (Bio-Rad, Hercules, CA, USA). Electrophoresis was carried out at a constant voltage of 200 V until the dye front reached the bottom of the gel. In-gel digestion and protein identification were performed using LC-MS/MS, either with the complete pool of proteins or by excising the most intense band at ~15 kDa. The detailed mass spectrometry procedures were conducted as previously described.

### Negative staining microscopy of LiCl extracts

Liquid cultures of *F. duncaniae* at stationary phase and LiCl extracts were analyzed using the negative staining method. Three microliters of the nano-object suspension were deposited on an air glow-discharged carbon-coated grid for 1 min. The excess liquid was blotted, and the grid was rinsed with 2% w/v aqueous uranyl acetate. The grids were visualized at 120 kV with a Tecnai 12 Spirit transmission electron microscope (Thermo Fisher, New York, NY, USA) equipped with a K2 Base 4 k × 4 k camera (Gatan, Pleasanton, CA, USA). Magnification was at 4400, 6500, or 15,000 x, corresponding to a pixel size at the specimen level of 0.83, 0.55, and 0.25 nm, respectively. Image analysis and dimensions calculations were obtained with ImageJ.^35^

### Recombinant MAM purification and antibody production

The plasmid pSTABY:*mam*, conferring ampicillin resistance, was introduced into *E. coli* BL21 (DE3) by transformation. The bacteria were grown in Luria–Bertani medium at 37°C, and the expression of the recombinant MAM (R-MAM) protein was induced using 1 mm isopropyl-β-D-thiogalactopyranoside (IPTG, Sigma) when the OD of the *E. coli* culture reached 0.6–0.8. Purification of R-MAM was conducted under denaturing conditions (8 M urea, with 50 mm imidazole, 100 mm sodium phosphate, and 10 mm Tris) utilizing Ni-NTA resin (Invitrogen) across a pH gradient ranging from 8.0 to 4.15. The eluate was subsequently collected and dialyzed against a dialysis buffer containing urea gradient (6 M, 4 M, 2 M, 1 M, 0 M) plus 50 mm TRIS, 1% glycerol, and 200 mm NaCl (pH 7.4) using a SpectraPor Dialysis Membrane (10000 MWCO, SpectrumLabs). This initial dialysis was performed for 5 h, and the urea was changed every 1 h. The dialysis process continued for an additional 24 h. Final dialysis was performed in 200 mL PBS 0.1 M plus glycerol 1%. The dialyzed MAM protein (15 μL) was analyzed via one-dimensional SDS-PAGE (12%), quantified using the BCA Protein Assay Reagent (Thermo Fisher Scientific) and subsequently sent to the GeneCust company for the polyclonal antibody production in rabbits. Anti-MAM antibody (1:1000) from *F. duncaniae* A2–165 was evaluated by Western Blot.

### Cryo-preparation and sectioning of bacterial cells

For ultrathin cryo-sectioning and immunogold labeling, bacterial cells were initially fixed with 4% paraformaldehyde (PFA) in culture media under oxygen-free conditions for 1 h. The cells were then transferred to an oxygen-containing environment, and the first fixative was removed. Next, another fixation step was conducted using a mixture of 4% PFA and 0.25% glutaraldehyde in 0.1 M phosphate buffer (pH 7.2) for 1 h, followed by another fixation step with 4% PFA for an additional hour. The cells were embedded in 2.8 M sucrose for cryoprotection and frozen rapidly in liquid nitrogen. Ultrathin cryosections were prepared according to the Tokuyasu method,^36^ optimized for preserving protein localization.

### Immunoelectron microscopy on bacteria cryosection

The grids were washed on 2% gelatin at 37°C to remove the embedding gelatin and remnants of the methylcellulose; then, they were quenched with glycine 50 mm in PBS, blocked with a buffer containing 1% BSA. Serum anti-MAM was incubated for 1 h in PBS/1% BSA/0.1% saponin; the grids were washed four times, then goat anti-rabbit IgG coupled to 10 nm colloidal gold particles (Aurion – Biovalley, France) and used at a 1/20 dilution in the presence of 0.1% saponin, for 30 min. The grids were again washed and post-fixed with 1% glutaraldehyde, and cryosections were embedded with 2% methylcellulose containing 4% uranyl acetate (4/1). Grids were examined with a Hitachi HT7700 electron microscope operated at 80 kV (Milexia – France), and images were acquired with a charge-coupled device camera (AMT). Three technical replicates were analyzed, and 30 bacterial cells were counted for labeling in each replicate.

### Statistical analyses

Statistical analyses were performed using GraphPad Prism software (version 8.0, GraphPad Inc., La Jolla, CA, USA). Data were analyzed for normality before selecting the appropriate statistical test. The specific statistical tests used for each analysis are indicated in the figure legends. P-values were reported as follows: *p* < 0.05; *p* < 0.01; *p < 0.001; **p < 0.0001.

## Results

### MAM peptides in the supernatant are diverse and increase during bacterial growth

MAM was initially identified by detecting seven specific peptides in the supernatant of *F. duncaniae* .^12^ To investigate this behavior more deeply, the dynamics of the production of MAM peptides during bacterial growth were evaluated by collecting supernatants at different stages of growth, from the early exponential to the late stationary phase. MAM peptides were recovered through solid phase extraction, followed by a 10 kDa filtration to avoid full-length proteins. LC-MS/MS analysis followed by MAM peptides identification revealed a notable increase in peptide abundance over time.

**Figure 1.**
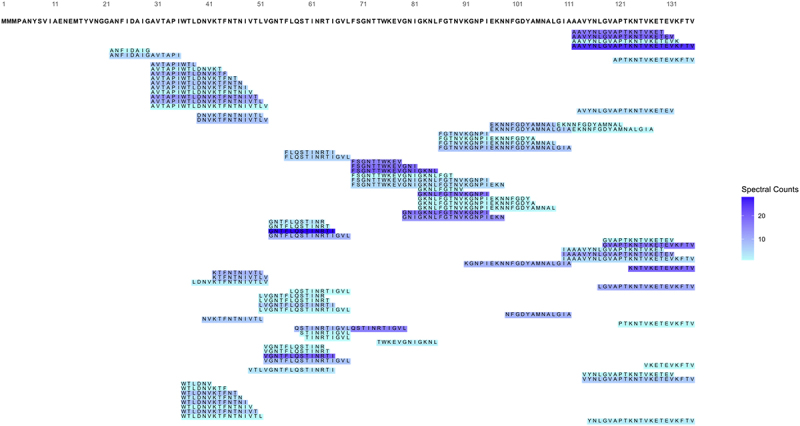
Peptide alignment map of MAM-identified peptides by LC-MS/MS peptidomic analysis. The figure shows the 86 unique MAM peptides identified by LC-MS/MS analysis. Each peptide is represented by a rectangle, with the peptide sequence within it overlapping with MAM’s amino acid sequence on the top. The rectangle’s color intensity indicates the peptide’s relative abundance, as measured by the number of spectral counts.

Our results demonstrated a great diversity of the detected MAM peptides (Figure 1). If we assemble all the peptides across the growth stages, it is possible to cover 84.4% of MAM’s total length. The identified peptides spanned from the 22^nd^ amino acid on the N-terminus to the C-terminus, covering almost the entire protein. Notably, the first 21 amino acids, from the initial methionine to the double glycine, were completely absent from our peptidomic analysis. We detected 88 peptide variants for the 135 amino acid-long MAM protein, varying in sequence, length, and abundance, with many differing by a single amino acid (Suplementary table 1). No indication of cleavage site by specific peptidases was observed, except for the putative signal peptide.

The identified peptides showed variable lengths, ranging from 5 to 29 amino acids long, with corresponding spectra counts that reflected their abundance. The most common peptide lengths were 12 and 13 amino acid residues. Notably, some peptides exhibited particularly high spectra counts, such as G_53_NTFLQSTINRTI_65_ (28 spectra) and V_52_GNTFLQSTINRTI_65_ (20 spectra), indicating their higher relative abundance. In between, a 23-amino-acid-residues-long peptide (A_112_AVYNLGVAPTKNTVKETEVKFTV_135_) that covers the C-terminus of MAM was also identified with an important abundance of 25 spectra (Supplementary data 1). The wide range in peptide sizes and dynamics in their relative abundance suggests a nonspecific cleavage process, likely due to degradation mechanisms rather than active secretion.

**Figure 2.**
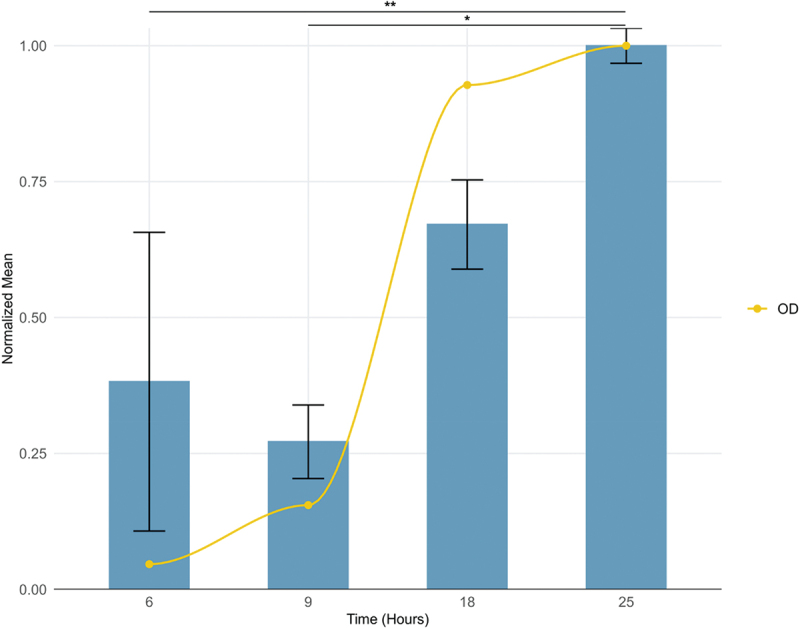
Dynamics of MAM peptides across different growth phases of *F. duncaniae*. Normalized mean number of MAM peptides identified at different growth phases: 6 h, 9 h (exponential phase), 18 h (early stationary phase), and 25 h (late stationary phase). Bar heights are scaled relative to the maximum peptide count to allow comparison with the OD curve. Actual normalized peptide values are provided in the main text and Supplementary table 2. The yellow solid line represents OD over time. Statistical significance was determined using Dunn’s multiple comparisons test, with significant differences indicated by asterisks (*p < 0.05, **p < 0.01).

During the exponential phase (6 and 9 h), an average of 1.25 ± 0.96 and 2.75 ± 0.50 peptides were detected, corresponding to normalized values of 26.25 ± 18.87 and 18.66 ± 4.64 peptides per OD, respectively. This number significantly increased to 41.25 ± 3.50 peptides (normalized to 46.12 ± 5.64 per OD) in the early stationary phase (18 h) and reached an average of 66.5 ± 2.38 peptides (normalized to 68.73 ± 2.23 per OD) in the late stationary phase (25 h). For visual clarity, the normalized peptide values shown in Figure 2 are scaled relative to the maximum value (Supplementary table 2).

### MAM is the most abundant envelope protein in the proteome of F. duncaniae

**Figure 3.**
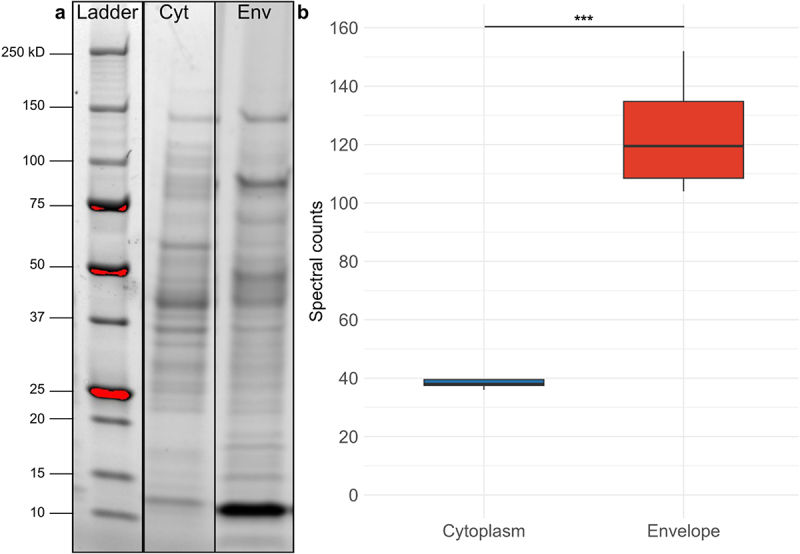
Abundance and localization of MAM in cytoplasmic and envelope fractions of *F. duncaniae* (a) SDS-PAGE gel showing protein gel ladder on the right, following by bands of cytoplasmic (cyt) and envelope (env) fractions. (b) Box plot of spectral counts representing MAM abundance in cytoplasmic (blue) and envelope (red) fractions. The box plot shows the median, interquartile range, and distribution. Statistical significance was determined using an unpaired t-test (for normal data) and F-test (for variance comparison). Significant differences are indicated as follow: ***p < 0.001.

While our results regarding MAM peptides in the supernatant suggested degradation processes, we aimed to investigate MAM’s presence in other subcellular localizations. *F. duncaniae* cultures were grown to the early stationary phase (18 h, OD ~ 1.6406) and fractionated into cytoplasmic and envelope samples through cell lysis and centrifugation (Figure 3(a)). Then, the samples were subjected to in-gel digestion and protein identification via LC-MS/MS. PCA analysis demonstrated a clear separation between cytoplasmic and envelope samples (Supplementary figure S2). The plot indicates that the first two principal components (Axis1 and Axis2) account for 43.2% and 15.6% of the variance, respectively. The separation of the cytoplasmic samples and envelope samples is evident along these axes, thus confirming the robustness of our fractionation process.

MAM represents 1.15% of the envelope proteome of *F. duncaniae*, making it the most abundant protein in this fraction, as indicated by the NSAF (Normalized Spectral Abundance Factor). When combining cytoplasmic and envelope fractions, MAM is the second most abundant protein in *F. duncaniae*, comprising approximately 0.76% of the total proteome. It is surpassed only by 3-hydroxyacyl-CoA dehydrogenase, NAD binding domain protein (HADH-NBD), the most abundant cytoplasmic protein. The average NSAF score for MAM in the envelope was 0.01146, compared to 0.0037 in the cytoplasm, indicating significant enrichment of MAM at the cell envelope (*p* < 0.05) (Figure 3(b), Supplementary data 2). The SDS-PAGE gel also corroborates these results, showing that the protein band at the level of 10–15 kDa (most probably MAM) is not only the most prominent one but also the more significantly abundant one in the envelope fraction, as compared to the cytoplasm. Interestingly, for the first time, with LC-MS/MS, we were able to identify the signal peptide contained in one of the four replicates of the insoluble extraction. The identified peptide is 42 amino-acid residues long (**M**_**1**_**MMPANYSVIAENEMTYVNGG**ANFIDAIGAVTAPIWTLDNVK_42_). It evidences a double glycine stretch at positions 20–21 and correlates to one spectrum count with 4.5 × 10^− 9^ E-value (Supplementary data 2). These results suggest that MAM plays a role in the envelope architecture of *F. duncaniae*.

### AlphaFold predictions reveal the structural features of MAM and its leader peptide

**Figure 4.**
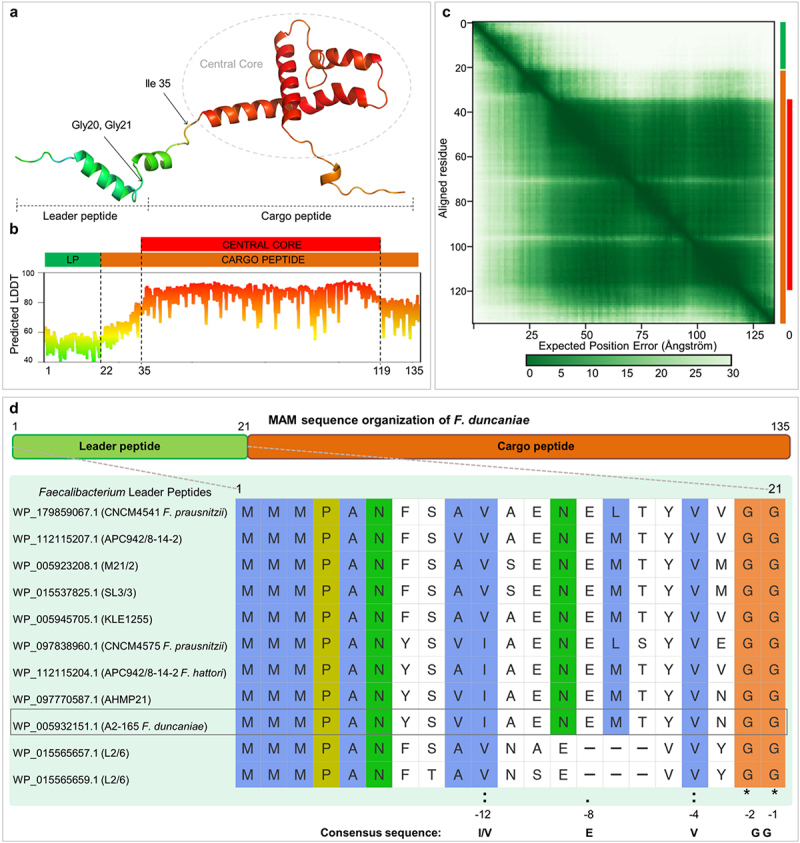
Schematic representation of MAM sequence and structure. (a) Predicted structure of the full-length MAM protein colored by pLDDT confidence scores. The N-terminus (LP, positions 1–35) has low confidence, while the central core (positions 37–70, 74–86, 100–119) shows higher confidence (red regions). (b) Schematic of MAM structure showing the leader peptide (LP, green), cargo peptide region (orange), and central core (red). The pLDDT graph aligns with the structural elements, indicating varying confidence levels. (c) PAE plot indicating structural blocks (1–22, 22–35, 36–135), with low intrablock and high interblock PAE. (d) Sequence schematic with leader peptide (green) and cargo peptide (orange). Below, sequence alignment of leader peptides from Faecalibacterium strains highlights conservation and consensus sequence.

The functional activity of proteins in their full-length (FL) or processed forms relies on their 3D structure, including when they harbor disordered segments. To get insight into MAM’s function, we sought to model its 3D structure, considering its two putative sizes, MAM full length (MAM-FL) and MAM excised of its leader peptide (MAM-∆LP). AlphaFold 3 (AF3) was employed to predict both 3D structures. Appropriately, the complete and truncated sequences of MAM were submitted online to the server (https://alphafoldserver.com/). Overall, the models show a pTM (predicted template modeling) score of 0,63 for MAM-FL and 0,6 for MAM-∆LP, with 82% and 72% ranking scores for the best models, respectively, indicating that the folding predictions are reliable. The 3D shape of MAM-FL displays a central helical core composed of two orthogonal helices, from which *N*- and C-terminal ends both extend out (Figure 4(a)). The N-terminal extension displays an L-shape with a helical double turn between residues 12 and 17, and the C-terminal end is unfolded. MAM-∆LP, deprived of its N-terminal segment, does not exhibit any significant change. Markedly, the Foldseek server evidences that this fold is unique and absent from the protein data bank.^28^ The lowest confidence in the structure is observed at the N-terminal segment, between amino acid positions 1 and 35. The predicted local distance difference test (pLDDT) also shows that this segment could not be highly structured (Figure 4(b)). In addition, the “PAE” (Predicted aligned error) associated with the prediction allows us to identify three distinct structure blocks 1–22, 22–35, and 36–135, whose predicted relative positions to each other are uncertain, which would suggest a certain flexibility of these domains in relation to each other (Figure 4(c)). This trait, associated with an L-shape, supports its function as a leader peptide.^32^

From position 22 to its C-terminus, MAM presents a central core composed of three helices, which are located between residues 37–70, 74–86, and 100–119, respectively. The leader peptide was formerly assessed to contain a pattern [MMMPANx8/11VxGG]^11^ where the double glycine stretch (GG) is typical of a cleavage site recognized by a cysteine peptidase transporter (Figure 4(d)).

To investigate the co-occurrence of MAM and PCAT, we screened the entire non-redundant FASTA database of 780 billion sequences. Using Fuzzpro, we identified sequences containing either MMMPANx(8,11)VxGG or GIE[T/L][V/I]K as signature motifs for MAM and PCAT, respectively. This analysis retrieved 257 MAM-like proteins, all displaying the leader peptide motif at their N-terminal end. These proteins were distributed across 50 genomes (Supplementary table 3). Strikingly, MAM motifs associated with *Faecalibacterium* genomes accounted for 95.33% of the identified sequences, strongly supporting the hypothesis that PCAT is consistently associated with MAM proteins, being involved in extremely specific interaction mechanism of *Faecalibacterium*. This feature prompted us to investigate the capacity of MAM 1) to have its signal peptide recognized and excised by its cognate PCAT; 2) to be contained in the inner cavity of PCAT dimer; and 3) to self-assemble and oligomerize after the transfer.

### MAM is likely processed and transported by the PCAT to the cell envelope

Although displaying some substitution in amino-acid composition within the genus, the MAM’s N-terminal end is highly conserved among all the *Faecalibacterium* species registered to date (Figure 4(d)).^11^ Also, the strictly observed genetic co-occurrence of a PCAT transporter with MAM leads us to investigate the direct interaction between PCAT and MAM. Actually, the N-terminus segment describes a pattern of residues and positions with Ile/Val at position −12, Glu-Met at −8, −7, Val at −4, and Gly–Gly at −2, −1. This pattern has been previously shown to be the signature of a leader peptide and markedly the hallmark of all Gram-positive PCAT substrates, with the cleavage site nomenclature being −1 and +1 after the double Glycine stretch that pins it with Gly-2Gly-1 (Supplementary figure S3A). Thus, it is very likely that the N-terminal segment of MAM is a leader peptide, and MAM is a substrate of PCAT.^37^

**Figure 5.**
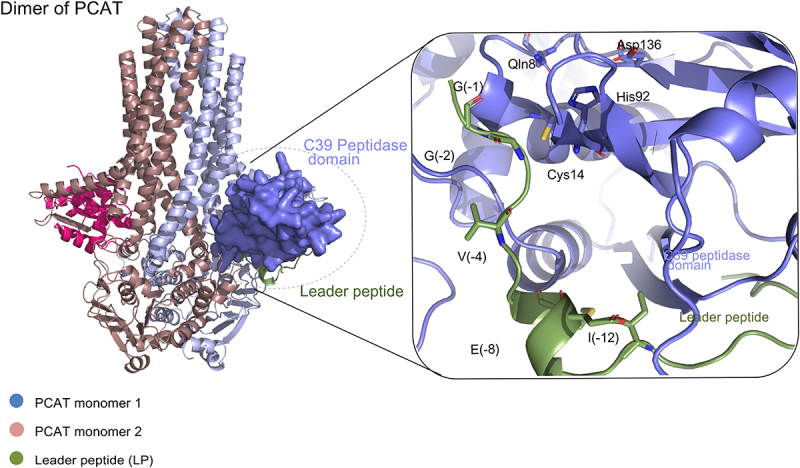
Interaction MAM leader peptide and the PCAT transporter. Predicted interaction of the MAM leader peptide (green) with the C39 peptidase domain (purple) of the PCAT. The PCAT transporter consists of two monomers: monomer 1 (purple) and monomer 2 (pink). Key catalytic residues in the peptidase domain are highlighted (Asp136, His92, and Cys14), along with stabilizing residues of the leader peptide: G(−1), G(−2), V(−4), E(−8), and I(−12). The transporter domain comprises transmembrane helices, with the inset zooming into the peptidase domain.

To further investigate this hypothesis, the molecular complex of MAM bound to the PCAT was modeled with AF3 and evaluated by Autodock. It revealed a high confidence zone in the position of MAM’s N-terminal segment when complexed to the C39 peptidase domain (Figure 5, Supplementary figure S3C). The molecular docking evaluated an interaction energy of −17.3 kcal.mol^−1^, evidence of a strong interaction between PCAT and MAM peptide. Also, the MAM peptide displays an L-shape conformation with the double GG at 4 Å and 6 Å from the catalytic residues Cys14 and His92, respectively. These features are expected to enable the PCAT substrate to accommodate the narrow groove of the peptidase domain. A close view of the binding shows that PCAT is also prime for MAM proteolysis. Cys14 is positioned as the nucleophile, with His92 oriented to polarize the attacking cysteine. Asp136 is placed to maintain His in an electronegativity and catalytically favorable position, while Gln8 is able to form an oxyanion hole that should stabilize the tetrahedral intermediate (Figure 5). Of note, those residues are homologous to Cys21, His99, Asp115, and Gln15 in PCAT1 of *A. thermocellus* ATCC27405, respectively. Finally, our model of PCAT in complex with MAM peptide was superimposed onto experimental PCAT1 bound to CtA substrate in *A. thermocellus*, with the superimposition restricted to PCAT αC (alpha-carbon) traces.^32,34^ Notably, MAM’s leader peptide was also superimposed on the crystal peptide at the peptidase site, with an RMSD (Root Mean Square Deviation) of 0.590 Å (Supplementary figure S3B). Similarly, the binding energy between the 23 residue CtA peptide and its PCAT1 in the cryo-EM solved structure^34^ was evaluated and showed a comparable affinity with 16.7 kcal.mol^−1^. This strongly suggests that the N-terminal segment could be a precursor peptide that leads MAM to be recognized and exported outside the cytoplasm, across the *F. duncaniae* envelope, by the PCAT, co-present in the genome, and thus highly susceptible to be the cognate transporter. Adequately, the mature form of MAM could be qualified as a cargo peptide, starting at Alanine 23 and ending at Valine 135, according to *F. duncaniae* sequence numbering.

To assess the importance of the double glycine motif for PCAT recognition and processing, we introduced targeted mutations in the MAM leader peptide, including deletion (ΔGG) and substitutions (GG→AA and GG→EE). Structural predictions using AF3 showed that these mutations significantly altered the interaction with the PCAT peptidase domain. In all tested mutations, the distances between the catalytic residues and the substrate (>5 Å) exceeded the typical range for stable interactions (~2.5–4.5Å).^38,39^ Therefore, they’re not compatible with effective molecular recognition, reinforcing the critical role of the GG motif in substrate recognition. (Supplementary Figure S4).

### Putative hexameric organization of MAM revealed by structural modeling after leader peptide removal

Proteins often become functional when assembled into supramolecular organizations rather than as isolated molecules.^40^ To investigate the physiological role of MAM, we examined its ability to self-organize into macromolecular structures and assessed the influence of its signal peptide on this arrangement. This was achieved by increasing the copy numbers of MAM-∆LP (lacking the leader peptide, corresponding to the amino acids 1–21) and MAM-FL, using AF3 to model complexes ranging from dimers (two units) to octamers (eight units). These structures and their associated metrics were thoroughly analyzed (Supplementary figure S5).

**Figure 6.**
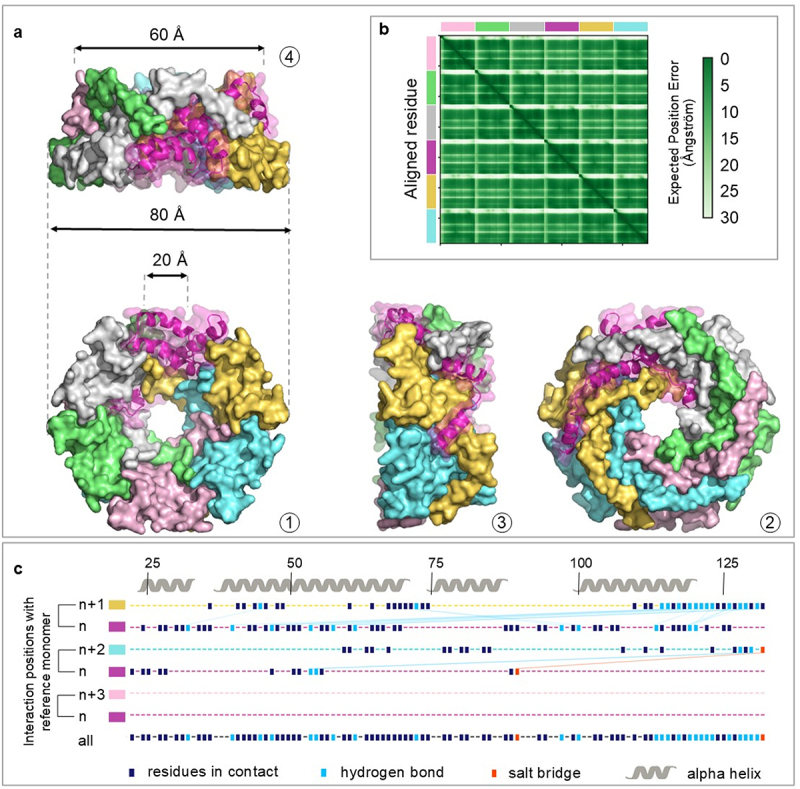
Predicted hexameric structure of MAM-∆LP. (a) Alphafold3-predicted hexameric structure of MAM-∆LP visualized in PyMOL, with subunits colored differently. Panels A-1 and A-2 show opposite faces of the hexamer, while A-3 and A-4 present the structural profile. The hexamer measures 80 Å in diameter on its widest face and 60 Å on the other face. The central hole is 20 Å in diameter. (b) PAE plot highlighting lower error at subunit interfaces (green blocks) and less confidence for peripheral regions. (c) schematic representation of amino acid interactions involved in the stability of the predicted MAM-∆LP hexamer structure, obtained with PISA. The upper part of the figure shows the amino acid numbers and the respective positions of the modeled α-helices. Bellow, each line corresponds to a subunit of the MAM-∆LP hexamer. Dark blue squares represent amino acid interaction points, light blue indicates hydrogen bonds, and orange indicates salt bridges. The first two lines display level 1 interactions (n, n+1), showing contacts between adjacent units. The next two lines correspond to level 2 interactions (n, n+2), indicating interactions between the second-adjacent subunit. The empty third line evidences the lack of level 3 interactions (n, n+3) corresponding to subunits on opposite sides. The last line represents all positions in interaction for one subunit.

The hexameric models of either MAM-FL or MAM-∆LP both reveal pore-forming structures with a comparable inner cavity of the buoy of 20 Å (Figure 6(a), Supplementary figure S6). Overall, the organization is the same, except for the external face of the buoy, where the N-terminal end is located. Nevertheless, both predicted complexes exhibited the same regular assembly concerning the position of each monomer. Despite this similarity in overall structure, MAM-FL exhibits external dimensions of the wider side of 100 Å, decreasing to 80 Å in MAM-∆LP due to the lack of the first 21aa. Considering the opposite surface, the diameter is about 60 Å for both structures. MAM-∆LP has a sensibly higher ranking score (0.94) than MAM-FL (0.74), suggesting better accuracy. This is likely due to the truncation of the N-terminal signal peptide in MAM-∆LP, which exhibits lower pLDDT and higher PAE values (Figure 6(b) and Supplementary figure S6). Indeed, the disordered level was around 10% lower in MAM-∆LP compared to MAM-FL. The interface-predictable Template Modeling (ipTM) score, calculated by AF3, was higher on the MAM-∆LP (78%) in comparison to the complete structure MAM-FL (54%). MAM-FL has a molecular weight of 86.9 kDa and is composed of 810 amino acids, whereas MAM-∆LP has a molecular weight of 73 kDa and consists of 684 amino acids. The theoretical pI of MAM-FL is 6.59, compared to a higher pI of 9.35 for MAM-∆LP. The number of negatively charged residues (Asp + Glu) is reduced from 54 in MAM-FL to 42 in MAM-∆LP, while the number of positively charged residues (Arg + Lys) remains constant at 54 for both hexamers. Fractional disorder is higher in MAM-FL (0.39) compared to MAM-∆LP (0.31) (Supplementary figure S5B). Altogether, these features emphasize that MAM-∆LP is more stable as a supramolecular assembly than MAM-FL.

The characteristics of the interface, measured by PISA, revealed that the contact area between the six chains for MAM-FL has an average of 1744.1 Å^2^, with a Gibbs free energy (ΔG) associated evaluated at −27.1 kcal.mol^−1^. In MAM-ΔLP, the contact area is 1695.5 Å^2^ with a Gibbs free energy unchanged at −26.2 ΔG kcal.mol^−1^, one more time evidencing that the N-terminal segment does not add much to the stability of the hexamer (Supplementary Table S4). Moreover, PISA results highlighted a broad and intricate interconnectivity between all the subunits of the complex. Notably, the interface is higher in the hexameric conformation, compared to all the modeled complexes. Within the hexamers, the surface area is slightly larger in MAM-FL (5012 Å^2^) as compared to MAM-∆LP (4448.9 Å^2^). The analysis reveals that hydrogen bonds (H-bonds) and salt bridges strongly mediate residue’s connectivity and structural arrangement (Figure 6(c)). Next, we augmented the number of copies and found that the circular arrangement is maintained in heptamers and octamers, with ranking scores of 0.94 and 0.89, respectively. This suggests comparable, if not better, stability for the hexamer form. Overall, these findings indicate a particularly stable and favorable edifice for MAM-∆LP in its hexameric conformation (Supplementary Figure S5B).

Calculations of electrostatic potential were performed on MAM-∆LP and MAM-FL hexamers, using the PyMOL APBS plugin, to investigate the distribution and contribution of charged residues. This analysis revealed distinct electrostatic profiles between the inner and outer protein surfaces. The wider surface, which corresponds to the central region of the amino-acid sequence, exhibits a predominance of positive charges spread across the surface, with interspersed negative charges. On this side, the central pore has a dominant negative potential, which might facilitate interactions or passages of positively charged ions or molecules. On the opposite side, which comprises the N-terminus residues (closer to the outer edge), the surface displayed more neutral charges. White regions dominate this surface side, indicating charge neutrality, particularly around the C-terminus, which forms the central pore. This observation is consistent with the role of envelope proteins, where different surfaces may interact with the bacterial membrane on one side while interacting with other extracellular or cytoplasmic components on the other. Such observations are suitable for both MAM-∆LP and MAM-FL (Supplementary Figure S7).

### LiCl extracts highlight MAM predominance and organized structural patterns

**Figure 7.**
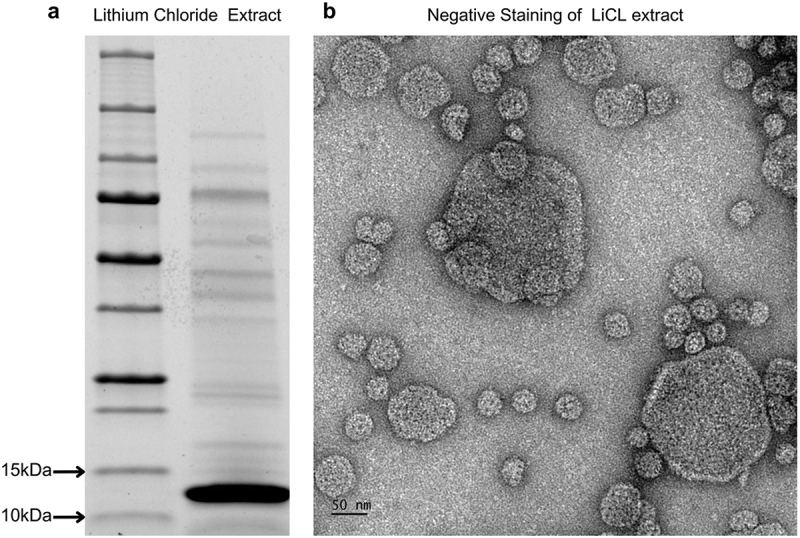
Proteomic Analysis of LiCl-extracted fraction. (a) SDS-PAGE electrophoresis of the LiCl-extracted fraction. The first lane contains the protein ladder, and the second lane contains the S-layer extract. (b) The negative staining microscopy of LiCl extracts reveals a complex landscape of circular fragments with varying diameters.

LiCl is often used to extract surface-associated proteins and cell envelope proteinases.^41^ To further investigate the association of MAM with the bacterial cell surface, the LiCl-extracted fraction from *F. duncaniae* was analyzed by SDS-PAGE electrophoresis (Figure 7(a)). Overall, fewer bands were observed at this extraction than at the previous cell envelope analysis. Notably, the band between 10 and 15 kDa was the most intense in the lane, likely corresponding to MAM, as its size aligns with MAM after processing the leader peptide (12,1 kDa). The complete protein pool was submitted for proteomic identification. This analysis revealed MAM as the most abundant protein based on the NSAF score (0,0188) (Supplementary figure S8 and Supplementary data 3). In-depth proteomic analysis of the extract resulted in MAM identification with log(E-value) of −474.10, identifying peptides covering 85.19% of the full-length protein, with only the first 21 amino acids missing. The analysis identified 203 spectra, all of which were specific, covering 49 sequences and 90 unique peptide modifications. In addition, the most intense band from a separate replicate was extracted for targeted protein identification and confirmed to be MAM (Supplementary table S5). These comprehensive results strongly indicate the predominance of MAM on the envelope of *F. duncaniae*.

In addition to identifying MAM as the predominant protein in the LiCl extracts, we sought to investigate the structural organization of these extracts using negative staining microscopy. Extracted fragments appeared as larger circular structures composed of smaller aggregates with similar organized patterns, exhibiting dark points and circular borders (Figure 7(b)). The diameters of the circular fragments were diverse, ranging from 29 nm to 220 nm. These observations are consistent with the predicted pore-like organization of hexameric assemblies but require further confirmation to establish a direct relationship. Together, these findings suggest that MAM may contribute to the organized structural motifs observed microscopically, consistent with its predicted hexameric assembly.

### Hexameric lattice observed in surface structures of F. duncaniae by in situ microscopy

**Figure 8.**
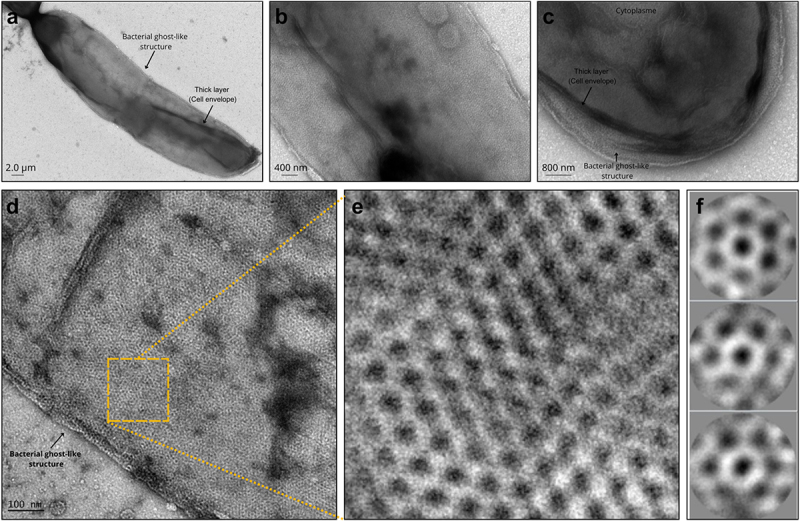
Electron microscopy of F. duncaniae cells. (a) Full cell of F. duncaniae with signs of internal degradation. (b) Section of the cell envelope showing an organized, layered structure. (c) Close-up of the cell end, highlighting a thick cell envelope and organized coat structure. (d) Structure resembling a bacterial ghost with an intact coat but absent internal contents. (e) High-resolution microscopy of (d), showing hexameric patterns. (f) SPA transform images demonstrate the hexameric arrangement, confirming six-fold symmetry..

Electron-microscopy imaging proves to be extremely helpful in order to obtain structural and functional information about cells and proteins.^42^ Here, *in situ*, negative stain electron microscopy of *F. duncaniae* cells revealed the bacilli expected shape of *F. duncaniae*. The internal cell content is surrounded by a thick, dark layer, likely representing the cell envelope. Some images showed regular cell shapes, while others showed their internal content shrinking or lost, likely due to cell lysis from oxygen exposure (Figure 8(a-d)). This led to the appearance of a remaining shield similar to bacterial ghosts, highlighting a structured layer with a regular pattern. This ordered lattice is visible at the outermost level. Circular structures with clear surroundings and dark centers indicate depressions, suggesting porosity.

To further evaluate this structure, Single Particle Analysis (SPA) of the bacterial lattice revealed a highly ordered hexameric structure. This periodic arrangement indicates the strong symmetry and regularity inherent in the protein assembly. The SPA images provided a detailed view of this hexameric pattern, consistently showing each central hexamer surrounded by six adjacent units, forming a macrostructure with six-fold symmetry. These units exhibited well-defined boundaries and central depressions, likely representing a porous layer. Quantitative measurements of these hexamers indicated an internal diameter with a mean of 14.7 nm (147 Å), varying from 12.5 to 17.4 nm in diameter. Moreover, the lattice organization suggests flexible properties, which are observed as curvatures (Figure 8(e-f)). This flexibility may significantly affect the protein complexes’ biological functionality and mechanical stability in the 3D bacterial conformational dynamics. These hexameric assemblies observed in the bacterial lattice are consistent with the predicted hexameric organization of MAM, as described previously.

### Immunogold labeling confirms MAM localization at the cell envelope poles

**Figure 9.**
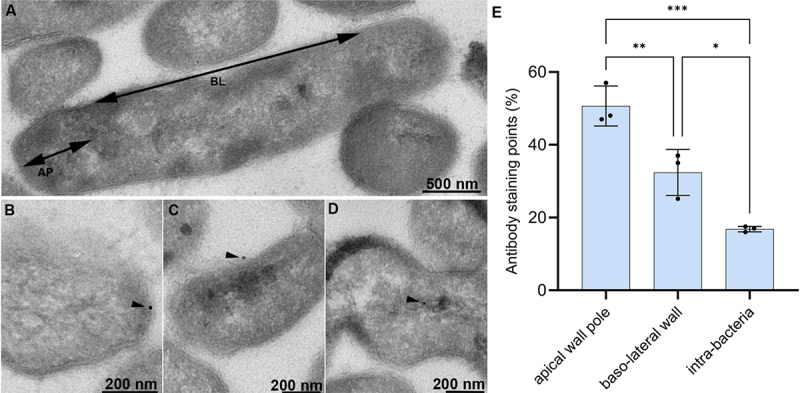
Immunolabeling of MAM in *F. duncaniae* cell. (a–d) Transmission electron microscopy (TEM) images of ultrathin sections of *F. duncaniae* cells showing the localization of MAM through immunogold labeling. Black dots represent the gold particles, indicating the presence of MAM. (a) TEM image of *F. duncaniae* illustrating the basolateral (BL) and apical (AP) regions of the bacteria; (b) Immunogold labeling localized at the apical pole; (c) basolateral side; and (d) intra-bacteria. (e) Percentage of labeling dots in each cell region, highlighting the higher significant labeling signal in the apical wall pole. Dots in the error bar indicate the three used technical replicates. Statistical analysis was performed via ANOVA followed by Tukey’s multiple comparisons post-test Significant comparisons are indicated: ***p < 0.001, **p < 0.01, and *p < 0.05..

Immunoelectron microscopy of ultrathin cryosections of *F. duncaniae* cells revealed the localization of MAM within the bacterial organization. Bacterial cells were sectioned and labeled with polyclonal anti-MAM antibodies, followed by secondary antibodies conjugated to 10 nm colloidal gold particles for visualization. Gold particles were predominantly observed at the cell periphery, indicating a strong association of MAM with the envelope layer (Figure 9(a-d), Supplementary figure S10). This peripheral distribution of MAM was consistent across multiple sections and different fields of view, confirming that MAM is localized mainly, more than 50%, at the outermost layer of the bacterial cell (Figure 9(e)). In contrast, minimal, less than 20%, gold labeling was detected in the cytoplasmic region, reinforcing the hypothesis that MAM is rather surface-associated. The electron-dense gold particles were distributed in a pattern that suggests MAM may form organized structures, potentially contributing to the integrity and functionality of the cell envelope.

## Discussion

*F. duncaniae* is a dominant species in the gut microbiota of healthy adults, known for its unique capacity to produce MAM, a protein with significant anti-inflammatory properties.^11^ However, the molecular characteristics and physiological role of MAM have not been well documented until now. Here, we reported *in vitro*, that MAM is the first protein, ranked by abundance, and primarily localized in the envelope fraction of *F. duncaniae*. We also demonstrated, *in silico*, compelling evidence that MAM could be the substrate of the adjacent PCAT, which is also remarkably expressed.^11^ We mark here that PCAT translocate MAM out, to the cell envelope. MAM could be recognized with its N-terminal leader peptide to be excised by the adjacent, and possibly cognate, PCAT. Moreover, MAM evidence *in silico* intrinsic ability to self-arrange into a hexameric complex, hence creating a pore-like supramolecular structure, secured by an intensive network of hydrogen bonds and salt bridges. In line with that, we reported, using microscopy techniques, the detection of a hexameric edifice with physical characteristics compatible with our *in silico* predictions and immunogold labeling of MAM, especially localized at the envelope of cell poles. These novel conclusions emphasize that MAM could shape the cell envelope and bolster *F. duncaniae*’s cell integrity while mediating interactions between the bacterial internal cell, the environment, and the host.

MAM’s known anti-inflammatory properties and beneficial effects observed in colitis mouse models have diverted investigations on MAM’s physiological and functional significance within the bacterium. MAM was primarily identified as peptides in the supernatant of *F. duncaniae* .^11^ Early structural predictions determined that MAM has more than 50% hydrophobic residues disseminated onto a putative globular organization. Low sequence complexity and intrinsic disorder were not predicted, and no transmembrane domain was correlated with MAM. Those earliest models were obtained by homology modeling, with the amino acid sequence indicating MAM having multiple β-sheet forming a core region flanked by α-helices.^12^ Ji-Hee Shin and collaborators also hypothesized on MAM tertiary structure, sharing features with the previously mentioned model.^13^ In addition, Auger et al. investigated the diversity of MAM based on genetic features and phylogenetic assays, highlighting MAM’s great phenotypic heterogeneity reflected in its anti-inflammatory potential.^11^ Hence, we aimed to investigate MAM’s physiological, structural, and functional features in *F. duncaniae*. Appropriately, we explore MAM traits using numerous methodological strategies to describe this protein extensively and assess its physiological role in the cell.

The first experimental identification of MAM was performed on the bacterial supernatant, which contains several fractions of MAM peptides, suggesting that MAM is a secreted protein.^12^ To confirm such behavior and the secretion dynamics of MAM peptides during the *F. duncaniae*’s growth, liquid culture supernatant was collected at different time steps, covering the bacterium’s growth from 6 to 25 h. This broad analysis revealed that MAM peptides are rarely detected in the early stages of growth, contrasting with a higher abundance at the stationary phase. Also, the peptides did not follow a specific pattern of secretion, neither in size nor in amino acid composition; many differed by just one amino acid from others. Such randomness is also observed in the first identified peptides. The four different peptides detected in the early stages of *F. duncaniae* growth (between 6 and 9 h) do not belong to a specific region but instead cover the middle-end of the protein (amino acid positions 52–65 and 112–135) (Figures 1 and 2). Markedly, none of them detects any N-terminal-containing peptides.

When comparing our results with those of the previous MAM identification performed by *Quévrain* et al.,^12^ a significant discrepancy in the number of identified MAM peptides is evident. While the earlier research identified only seven peptides using MALDI-TOF (Matrix-Assisted Laser Desorption/Ionization Time-of-Flight) and FT-ICR (Fourier Transform Ion Cyclotron Resonance) mass spectrometry, our approach identified 86 MAM peptides. Several factors could explain this difference. First, the sensitivity and the resolution of the Orbitrap Fusion Lumos Tribrid mass spectrometer employed in our study are superior, enabling largely comprehensive peptide detection and identification.^43,44^ Additionally, our methodological approach involved a more extensive fractionation process, with samples collected at various growth phases (6, 9, 18, and 25 h) and from four replicates, increasing the likelihood of detecting a broader range of peptides. Furthermore, using the X! Tandem software for data processing may have contributed to more robust peptide identification compared to the methodologies used in the previous study.^24,43^ This significant increase in the number of identified peptides highlights the effectiveness of our methodological enhancements and, importantly, highlights the complexity of MAM peptide and the abundance of MAM production in *F. duncaniae*.

This accumulation of MAM peptides in the supernatant during the stationary phase likely reflects a physiological response to nutrient limitation and stress, leading to increased bacterial lysis. Transcriptomic data show that mam expression remains high across growth phases,^14^ suggesting that peptide abundance does not correlate with gene expression levels or cell density, but rather with enhanced protein release due to cell damage. Environmental conditions such as low acetate availability or acidic pH may further contribute to stress and promote lysis-mediated release, although MAM production itself appears to be transcriptionally stable under low acetate conditions.^14,45^ Consequently, we hypothesize that MAM is not a secreted protein *per se* but is likely present in the supernatant due to a nonspecific degradation process, possibly cell death, lacking distinct cleavage sites. This is fully consistent with the capacity of MAM leader peptide to be recognized and cleaved off by PCAT, which strictly co-occurs and is located in the vicinity of MAM. This explains why the leader peptide/N-terminus is not detectable in our proteomic and peptidomics analysis. Notably, MAM is able to be embodied within the inner groove formed by the PCAT dimer.

The evidence that MAM is not actively secreted raises the question of its subcellular localization. Several cell fractionation procedures have been established, each adapted to specific bacterial envelope compositions.^41^ However, the cell envelope characteristics of *F. duncaniae* remain undescribed. Therefore, we employed a preliminary method to separate the envelope from cytoplasmic fractions and investigate MAM localization. Cell pellets were subjected to chemical and physical lysis, followed by high-speed centrifugation to separate the internal soluble (cytoplasmic fraction) from the envelope (insoluble fraction) content.^46,47^ Samples were then submitted to LC-MS/MS for protein identification and i2MassChroq^48^ was used for spectral counting quantification.

Our results assessed that MAM is one of the most abundant proteins in *F. duncaniae*’s proteome and the most abundant protein in the envelope fraction (Figure 3). Principal Component Analysis (PCA) demonstrates that our separation approach effectively differentiated cytoplasm from envelope proteins. Notably, the NSAF score, which normalizes spectral counts by protein length to account for differences in protein size, identified MAM as the most abundant protein in the envelope fraction. This normalization is particularly relevant for accurately comparing the relative abundance of proteins within complex mixtures.^49^ In addition, our findings suggest that the conventional cell fractionation method, which typically treats the envelope fraction as cellular debris, may not be optimal for *F. duncaniae*.^50^ This approach could hide critical insights into MAM’s subcellular localization and abundance. Moreover, *F. duncaniae* is a fragile bacterium that is difficult to cultivate due to its extreme oxygen sensitivity, presenting significant challenges during cell manipulation. While refined fractionation techniques exist, our approach provided sufficient resolution under experimental conditions, highlighting MAM as a key envelope component. However, these results also underscore the necessity of future investigations into *F. duncaniae*’s cell organization, with further optimization of cell fractionation methods specific to this bacterium.

MAM’s amino acid composition, genetic organization, structural conformation, and supramolecular assembling capacity were also investigated. MAM is exclusively produced by the genus *Faecalibacterium*. Other studies from the group have recently highlighted this singularity.^11^ Having such a distinctive sequence, no relevant homology with other proteins is observed, and no known domains were identified. The MAM sequence of *F. duncaniae* differs from the MAM of other *Faecalibacterium* species by up to 40%. The genomic region of MAM’s coding sequence harbors several proteins, including the PCAT.^11^

ABC transporters integrate a large group of integral membrane proteins present in a vast number of living organisms. They are composed of two Nucleotide-Binding Domains (NBD) responsible for the processing of ATP and two Transmembrane Domains (TMDs), which form the core of the active export of diverse substrates, including proteins, ions, lipids, and peptides.^51^ PCATs are unique members of the ABC transporter group. They are formed by two peptidases capable of transporting peptides and proteins across membranes and acting on the maturation process through their proteolytic activity. They act, for example, on the secretion of quorum-sensing and antimicrobial peptides.^15,52^ PCATs in Gram-positive bacteria are known to recognize and cleave off leader peptides that specifically display an L-shaped N-terminal segment with a double glycine stretch (GG) while being able to embody the cargo protein to be exported in a disordered shape. This step is next followed by transferring the remaining cargo protein outside the cytosol. In Gram-negative bacteria, PCATs are involved in a complex formation of membrane proteins in a secretion system Type I (T1SS). They compose a transport core between the inner and outer membranes; in this case, the proteolytic process is absent, resulting in the maintenance of the leader peptide.^32,37^

In this study, Alphafold3 was used to model the MAM structure as a monomer, followed by docking the MAM leader peptide in a complex with the ABC transporter, considered as a functional dimer. AlphaFold is considered the most accurate tool for structural predictions, with the added capability of predicting complex interactions.^26^ Although the sequence of MAM can be variable among different species of Faecalibacterium, three motifs recapitulate all MAM sequences. The predicted monomeric structure of MAM revealed a core region consisting of three alpha-helices connected by flexible coil regions with a potentially unfolded C-terminus (Figure 4(a)). Remarkably, the N-terminus is strictly conserved if we consider the [MMMPANx8/11VxGG] motif, and it has been shown that it is necessary and sufficient to retrieve MAM-like protein in a dataset of hundreds of millions of sequences.

Indeed, the structure prediction of MAM and docking of its 22-residue-long extended leader peptide into the PCAT active site highlight a strong interaction at the cysteine peptidase domain of the PCAT. The N-terminal segment of MAM evidences an L-shape feature, which is required to accommodate into the PCAT conserved active site and to prime the GG stretch for proteolysis. The process leads to the excision of the leader peptide, in which a consensus sequence has been previously identified in other organisms, also located at the N-terminus and formed by Ile/Leu/Val(−12)-(X)3-Glu(−8)-Leu(−7)-Val(−4)-Gly(−2)Gly(−1).^37,52^ Markedly, this pattern sticks to the MAM’s consensus sequence described above Leu(−12)-(X)3-Glu(−8)-Leu(−7)-Gly(−2)Gly(−1) (Figure 4(d) and Supplementary figure S3A). This observation strengthens the indication of PCATs as the cognate transporter of MAM.

These *in silico* interactions strongly correlate with those observed in the recently solved cryo-EM structure of the PCAT1 transporter bound to a 21-residue CtA substrate in *Clostridium thermocellum*.^53^ The conserved amino acid residues at the active site, the shared pattern of substrate binding, and similar interaction energies suggest that PCAT recognizes and cleaves the MAM leader peptide, acting as its cognate transporter. Supporting this hypothesis, the MAM leader peptide has been detected by LC-MS/MS only once, with one related spectra count. In contrast, the MAM cargo peptide has been consistently identified as a cell envelope component. This evidence indicates that MAM is likely a physiological substrate of PCAT, which processes and exports it to participate into the assembly of the *F. duncaniae* cell envelope (Figure 5).

The subcellular localization of MAM at the cell envelope, the absence of a strong secretion pattern, the high abundance and the putative transportation through PCAT led us to use AlphaFold again to investigate the capability of a structural super-organization of MAM. Starting from dimers and extending to octamers, our modeling indicated that MAM is likely arranged in a pore-organized structure, with the highest confidence attributed to the hexameric assembly of MAM cargo peptide (Figure 6). Moreover, the electrostatic potential map of the hexameric model indicates its charge distribution is suitable for interaction with the bilipid membrane (Supplementary figure S7).^54^ The presence of negative and positive charges across the protein’s surface suggests that MAM may interact with polar heads and hydrophobic tails of the lipid bilayer. This feature can act to facilitate protein anchoring and interactions and maintain the proper orientation of MAM within the membrane. These findings suggest that once exported to the cell envelope, after N-terminal cleavage by the PCAT, MAM would predominantly organize in a stable oligomeric complex, forming a pore with a central hole.

Recent microscopy techniques were applied to visualize and analyze *F. duncaniae* cells accurately. Our findings revealed significant insights into the cell envelope’s architecture and functionality. The in situ negative stain electron microscopy confirmed the bacilli shape of *F. duncaniae* and highlighted a porous bacterial coat. The observed shrinkage and cell lysis from oxygen exposure resulted in cells with the appearance of bacterial ghosts, which persisted possibly after cell lysis^55^(Figure 8(a-d)). Fast Fourier Transform and Single Particle Analysis revealed a crystalline pattern with six-fold symmetry by exhibiting a regular, hexameric lattice structure. The flexibility observed in the lattice’s curvature may contribute to its biological function and mechanical stability (Figure 8).

Among the different pore-organized proteins, MAM’s subcellular localization, diversity, the absence of structural homologs, it’s high abundance, extractability using standard S-layer extraction methods, such as LiCl and the putative propensity to self-assemble into pore-like structures as visualized by our microscopy assays, indicate a compatible role as an S-layer protein. For instance, *Haloferax volcanii* is an archaeon that has a protein with self-arrangement properties organized into hexameric or pentameric structures, forming the S-layer coat on the cell surface and the surface of exosomes.^56^ S-layer proteins from the bacteria *Deinococcus radiodurans* and the archaea *Sulfolobus acidocaldarius* also have the capacity to create hexamers, with the central pore structure. The assembly of several hexamers forms an ordered lattice, which provides structural integrity and protection to the organism.^57,58^ In beneficial bacterial species like *L. acidophilus,*^59^ *L. brevis,*^60^ *L. crispatus,*^61,62^ *L. helveticus*,^63,64^ and *P. freudenreichii*,^65^ S-layers were also identified, having beneficial properties to the host.^66^

After using LiCl, a common surface proteins extraction agent,^67,68^ through proteomic analysis, we obtained an enriched envelope sample in which MAM was identified as the most abundant protein. Negative staining microscopy of the LiCl extract supported the visualization of an ordered porous lattice, this time in circular fragments, but with the same pattern as the surrounding *F. duncaniae* cells. The hexameric structure of MAM modeled by AlphaFold matched the organization observed in these networks regarding the hexameric arrangement (Figures 6–8). These findings support the possibility that MAM could function as an S-layer protein. However, further research is necessary to confirm this, particularly to determine whether there is a complete proteinaceous bacterial coat or if MAM is a membrane-associated protein.

Immunogold labeling of *F. duncaniae* cells obtained with polyclonal antibodies raised against recombinant purified MAM revealed black dots on the peripheral bacterial surface, confirming the presence of MAM in the cell envelope (Figure 9).

Interestingly, MAM detection in *F. duncaniae* cells was more pronounced at the bacterial poles than in the mid-cell region (Figure 9(b)). Polar proteins are known to play roles in genome segregation, cell division, septum formation, signal transduction, and dynamic regulation, all of which are crucial for determining protein functionality.^69,70^ For example, the DivIVA protein in *Bacillus subtilis* localizes to the poles and septa, playing a key role in morphogenesis and cell division.^71^ *Caulobacter crescentus* exhibits polar proteins such as TipN, essential for cell polarity and proper organization of cell proteins and organelles,^72^ and PopZ, which anchors chromosomes and facilitates segregation.^73^ These proteins indeed have the capacity to form multimeric suprastructures; however, their assemblies do not typically resemble organized lattices or pore-like structures. Instead, they form unique higher-order assemblies arranged for their specific cellular functions.^74,75^ On the other hand, the biogenesis of S-layer protein, which is arranged in hexameric units in a porous lattice, was recently described in *Corynebacterium glutamicum*. The production occurs specifically in the cell poles, being compatible with cell elongation and the peptidoglycan synthesis site.^76^

These findings strengthen the hypothesis that MAM is essential for maintaining cell envelope integrity in addition to opening new avenues for exploring MAM’s potential role in the cell cycle. This localization might be linked to cell division, shape maintenance, or envelope remodeling. Improving purification and folding protocols, besides the combination of cryo-EM and immunogold labeling, are further steps to conduct and precisely determine MAM’s contribution to the cell integrity and cycle, and whether it can be classified as an S-layer protein.

Although the primary focus of this study is the characterization of MAM, it is important to note that its abundance, structure, and positioning on the cell surface could influence its recognition by cellular receptors and its ability to modulate host anti-inflammatory responses. While *F. duncaniae* effects have been primarily attributed to peptides identified in the supernatant, the present study suggests that MAM may mediate the direct interaction between the bacteria and the host. This interaction could help explain the beneficial effects of the bacterium, including its immunomodulatory actions, such as the inhibition of the NF-κB pathway and maintenance of barrier integrity.^11,16,18^ This information represents a significant advancement for future studies aimed at evaluating MAM’s therapeutic potential, as it may serve either as an integral component in bacterial-derived products or as a functional element in *F. duncaniae*-based interventions. Nonetheless, further research is necessary to determine whether MAM is the primary effector molecule responsible for the direct beneficial effects of *F. duncaniae*, as well as to clarify the associated molecular mechanisms.

## Supplementary Material

Supplemental Material

## Data Availability

The mass spectrometry proteomics data reported in this work have been deposited in the ProteomeXchange Consortium database (http://proteomecentral.proteomexchange.org) via the PRIDE partner repository, with the dataset identifier PXD057939.
