# Supplementary material for: The Microbial Anti-Inflammatory Molecule (MAM) is a key protein processed and exported to Faecalibacterium duncaniae envelope
Source: Gut Microbes. 2025 Jun 18;17(1):2519695. doi: 10.1080/19490976.2025.2519695 (PMC12184118; doi:10.1080/19490976.2025.2519695)
Supplement: Supplemental Material [file KGMI_A_2519695_SM5476.zip › Supplementary material.docx]

**Supplementary material**

**Supplementary table 1.** Information about the identified peptides in the supernatant

| **Peptide** | **UniProt id** | **Start position** | **End position** | **Spectra count** |
| --- | --- | --- | --- | --- |
| AAVYNLGVAPTKNTVKET | C7H4X2 | 112 | 129 | 17 |
| AAVYNLGVAPTKNTVKETEV | C7H4X2 | 112 | 131 | 20 |
| AAVYNLGVAPTKNTVKETEVK | C7H4X2 | 112 | 132 | 2 |
| AAVYNLGVAPTKNTVKETEVKFTV | C7H4X2 | 112 | 135 | 25 |
| ANFIDAIG | C7H4X2 | 22 | 29 | 1 |
| ANFIDAIGAVTAPI | C7H4X2 | 22 | 35 | 6 |
| APTKNTVKETEVKFTV | C7H4X2 | 120 | 135 | 4 |
| AVTAPIWTL | C7H4X2 | 30 | 38 | 8 |
| AVTAPIWTLDNVKT | C7H4X2 | 30 | 43 | 3 |
| AVTAPIWTLDNVKTF | C7H4X2 | 30 | 44 | 10 |
| AVTAPIWTLDNVKTFNT | C7H4X2 | 30 | 46 | 5 |
| AVTAPIWTLDNVKTFNTN | C7H4X2 | 30 | 47 | 8 |
| AVTAPIWTLDNVKTFNTNI | C7H4X2 | 30 | 48 | 6 |
| AVTAPIWTLDNVKTFNTNIV | C7H4X2 | 30 | 49 | 2 |
| AVTAPIWTLDNVKTFNTNIVT | C7H4X2 | 30 | 50 | 13 |
| AVTAPIWTLDNVKTFNTNIVTL | C7H4X2 | 30 | 51 | 10 |
| AVTAPIWTLDNVKTFNTNIVTLV | C7H4X2 | 30 | 52 | 1 |
| AVYNLGVAPTKNTVKETEV | C7H4X2 | 113 | 131 | 5 |
| DNVKTFNTNIVTL | C7H4X2 | 39 | 51 | 7 |
| DNVKTFNTNIVTLV | C7H4X2 | 39 | 52 | 8 |
| EKNNFGDYAMNAL | C7H4X2 | 96 | 108 | 8 |
| EKNNFGDYAMNAL | C7H4X2 | 96 | 108 | 2 |
| EKNNFGDYAMNALGIA | C7H4X2 | 96 | 111 | 4,5 |
| FGTNVKGNPI | C7H4X2 | 86 | 95 | 6 |
| FGTNVKGNPIEKNNFGDYA | C7H4X2 | 86 | 104 | 1 |
| FGTNVKGNPIEKNNFGDYAMNAL | C7H4X2 | 86 | 108 | 5 |
| FGTNVKGNPIEKNNFGDYAMNALGIA | C7H4X2 | 86 | 111 | 8 |
| FLQSTINRTI | C7H4X2 | 56 | 65 | 6 |
| FLQSTINRTIGVL | C7H4X2 | 56 | 68 | 6 |
| FSGNTTWKEV | C7H4X2 | 69 | 78 | 16 |
| FSGNTTWKEVGNI | C7H4X2 | 69 | 81 | 18 |
| FSGNTTWKEVGNIGKNL | C7H4X2 | 69 | 85 | 20 |
| FSGNTTWKEVGNIGKNLFGT | C7H4X2 | 69 | 88 | 1 |
| FSGNTTWKEVGNIGKNLFGTNVKGNPI | C7H4X2 | 69 | 95 | 5 |
| FSGNTTWKEVGNIGKNLFGTNVKGNPIEKN | C7H4X2 | 69 | 98 | 5 |
| GKNLFGTNV | C7H4X2 | 82 | 90 | 2 |
| GKNLFGTNVKGNPI | C7H4X2 | 82 | 95 | 18 |
| GKNLFGTNVKGNPIEKNNFGDY | C7H4X2 | 82 | 103 | 1 |
| GKNLFGTNVKGNPIEKNNFGDYA | C7H4X2 | 82 | 104 | 2 |
| GKNLFGTNVKGNPIEKNNFGDYAMNAL | C7H4X2 | 82 | 108 | 2 |
| GNIGKNLFGTNVKGNPI | C7H4X2 | 79 | 95 | 18 |
| GNIGKNLFGTNVKGNPIEKN | C7H4X2 | 79 | 98 | 14 |
| GNTFLQSTINR | C7H4X2 | 53 | 63 | 4 |
| GNTFLQSTINRT | C7H4X2 | 53 | 64 | 2 |
| GNTFLQSTINRTI | C7H4X2 | 53 | 65 | 28 |
| GNTFLQSTINRTIGVL | C7H4X2 | 53 | 68 | 10 |
| GVAPTKNTVKETEV | C7H4X2 | 118 | 131 | 2 |
| GVAPTKNTVKETEVKFTV | C7H4X2 | 118 | 135 | 18 |
| IAAAVYNLGVAPTKNTVKET | C7H4X2 | 110 | 129 | 4 |
| IAAAVYNLGVAPTKNTVKETEV | C7H4X2 | 110 | 131 | 12 |
| IAAAVYNLGVAPTKNTVKETEVKFTV | C7H4X2 | 110 | 135 | 5 |
| LDNVKTFNTNIVTLV | C7H4X2 | 38 | 52 | 3 |
| LGVAPTKNTVKETEVKFTV | C7H4X2 | 117 | 135 | 11 |
| LQSTINRTIGVL | C7H4X2 | 57 | 68 | 1 |
| LVGNTFLQSTINR | C7H4X2 | 51 | 63 | 3 |
| LVGNTFLQSTINRT | C7H4X2 | 51 | 64 | 1 |
| LVGNTFLQSTINRTI | C7H4X2 | 51 | 65 | 7 |
| LVGNTFLQSTINRTIGVL | C7H4X2 | 51 | 68 | 2 |
| KGNPIEKNNFGDYAMNALGIA | C7H4X2 | 91 | 111 | 10 |
| KNTVKETEVKFTV | C7H4X2 | 123 | 135 | 19 |
| KTFNTNIVTL | C7H4X2 | 42 | 51 | 10 |
| KTFNTNIVTLV | C7H4X2 | 42 | 52 | 8 |
| NFGDYAMNALGIA | C7H4X2 | 99 | 111 | 10 |
| NVKTFNTNIVTL | C7H4X2 | 40 | 51 | 7 |
| PTKNTVKETEVKFTV | C7H4X2 | 121 | 135 | 1 |
| QSTINRTIGVL | C7H4X2 | 58 | 68 | 12,5 |
| STINRTIGVL | C7H4X2 | 59 | 68 | 2 |
| TINRTIGVL | C7H4X2 | 60 | 68 | 1 |
| TWKEVGNIGKNL | C7H4X2 | 74 | 85 | 3 |
| VGNTFLQSTINR | C7H4X2 | 52 | 63 | 2 |
| VGNTFLQSTINRT | C7H4X2 | 52 | 64 | 2 |
| VGNTFLQSTINRTI | C7H4X2 | 52 | 65 | 20 |
| VGNTFLQSTINRTIGVL | C7H4X2 | 52 | 68 | 8 |
| VKETEVKFTV | C7H4X2 | 126 | 135 | 1 |
| VTLVGNTFLQSTINRTI | C7H4X2 | 49 | 65 | 4 |
| VYNLGVAPTKNTVKETEV | C7H4X2 | 114 | 131 | 2 |
| VYNLGVAPTKNTVKETEVKFTV | C7H4X2 | 114 | 135 | 7 |
| WTLDNV | C7H4X2 | 36 | 41 | 3 |
| WTLDNVKTF | C7H4X2 | 36 | 44 | 1 |
| WTLDNVKTFNT | C7H4X2 | 36 | 46 | 8 |
| WTLDNVKTFNTN | C7H4X2 | 36 | 47 | 4 |
| WTLDNVKTFNTNI | C7H4X2 | 36 | 48 | 8 |
| WTLDNVKTFNTNIV | C7H4X2 | 36 | 49 | 1 |
| WTLDNVKTFNTNIVT | C7H4X2 | 36 | 50 | 7 |
| WTLDNVKTFNTNIVTL | C7H4X2 | 36 | 51 | 1 |
| YNLGVAPTKNTVKETEVKFTV | C7H4X2 | 115 | 135 | 3 |

**Supplementary table 2.** Raw and normalized values for OD_600_ and peptide quantification over time.

| **Time (H)** | **Mean O.D.** | **Normalized O.D.** | **Real Peptides (mean)** | **Mean Peptides per O.D.** | **Normalized Peptides** | **SD Peptides per OD** |
| --- | --- | --- | --- | --- | --- | --- |
| 6 | 0.045 | 0.0465 | 1.25 | 26.25 | 0.382 | 18.87 |
| 9 | 0.150 | 0.1550 | 2.75 | 18.66 | 0.271 | 4.64 |
| 18 | 0.8975 | 0.9280 | 41.25 | 46.12 | 0.671 | 5.64 |
| 25 | 0.9675 | 1.0000 | 66.50 | 68.73 | 1.000 | 2.23 |

**Supplementary table 3.** List of genomes harboring the motif MMMPANx(8,11)VxGG in the nr fasta database and the number of proteins associated

| **Specie** | **Nº of associated proteins** |
| --- | --- |
| *Candidatus Faecalibacterium avium* | 1 |
| *Candidatus Faecalibacterium faecigallinarum* | 1 |
| *Candidatus Faecalibacterium gallistercoris* | 1 |
| *Candidatus Faecalibacterium intestinavium* | 1 |
| *Candidatus Faecalibacterium intestinigallinarum* | 1 |
| *Candidatus Faecalibacterium intestinipullorum* | 1 |
| *Faecalibacterium* | 16 |
| *Faecalibacterium butyricigenerans* | 2 |
| *Faecalibacterium duncaniae* | 4 |
| Faecalibacterium faecis | 1 |
| *Faecalibacterium gallinarum* | 1 |
| *Faecalibacterium hattorii* | 1 |
| *Faecalibacterium hominis (ex Afrizal et al. 2022)* | 1 |
| Faecalibacterium intestinale | 1 |
| *Faecalibacterium longum* | 3 |
| *Faecalibacterium prausnitzii* | 137 |
| *Faecalibacterium sp.* | 35 |
| *Faecalibacterium sp. AF10-46* | 1 |
| *Faecalibacterium sp. AF27-11BH* | 1 |
| *Faecalibacterium sp. AM43-5AT* | 1 |
| *Faecalibacterium sp. An121* | 1 |
| Faecalibacterium sp. An122 | 1 |
| *Faecalibacterium sp. An192* | 1 |
| *Faecalibacterium sp. An58* | 1 |
| *Faecalibacterium sp. An77* | 1 |
| *Faecalibacterium sp. BCRC 81149* | 1 |
| *Faecalibacterium sp. DFI.5.82* | 1 |
| Faecalibacterium sp. HTF-128 | 1 |
| Faecalibacterium sp. i21-0019-B1 | 1 |
| *Faecalibacterium sp. I2-3-92* | 1 |
| *Faecalibacterium sp. I3-3-33* | 1 |
| *Faecalibacterium sp. I3-3-89* | 2 |
| *Faecalibacterium sp. I4-3-84* | 1 |
| *Faecalibacterium sp. IP-3-29* | 1 |
| *Faecalibacterium sp. Marseille-P9590* | 2 |
| *Faecalibacterium sp. Marseille-Q3530* | 1 |
| *Faecalibacterium sp. Marseille-Q4137* | 1 |
| *Faecalibacterium sp. Marseille-Q4896* | 1 |
| *Faecalibacterium sp. OF04-11AC* | 2 |
| *Faecalibacterium sp. OM04-11BH* | 1 |
| Faecalibacterium taiwanense | 1 |
| Faecalibacterium tardum | 1 |
| *Nitrososphaerales archaeon* | 1 |
| *Nitrososphaerota archaeon* | 2 |
| *Oscillospiraceae bacterium* | 5 |
| *Ruminococcaceae bacterium* | 2 |
| *Shigella flexneri* | 1 |
| *Streptococcus infantis* | 1 |
| *unclassified Faecalibacterium* | 3 |
| *uncultured Faecalibacterium sp.* | 6 |

**Supplementary table 4:** Interfaces in the hexameric structures of MAM-FL and MAM-∆LP

The table includes the interface identifier (NN), selection range (Range), number of interfacing atoms (iNat), number of interfacing residues (iNres), total solvent-accessible surface area (Surface, Å²), interface area (Å², calculated as half the difference in accessible surface areas of isolated and interfacing structures), solvation free energy gain (ΔiG, where negative values indicate hydrophobic interfaces or positive protein affinity), ΔiG P-value (significance of solvation free energy gain), number of potential hydrogen bonds (NHB), salt bridges (NSB), and disulfide bonds (NDS) across the interface.

| **MAM-FL** | | **Structure 1** | | | | **Structure 2** | | | |  |  |  |  |  |  |
| --- | --- | --- | --- | --- | --- | --- | --- | --- | --- | --- | --- | --- | --- | --- | --- |
| **Id** | NN | **Range** | **iNat** | **iNres** | **Surface Å2** | **Range** | **iNat** | **iNres** | **Surface Å2** | **Interface area, Å2** | **ΔiG**  **kcal/mol** | **ΔiG**  **P-value** | **N_HB_** | **N_SB_** | **N_DS_** |
| 1 | 1 | A | 175 | 41 | 11709 | F | 189 | 53 | 11702 | 1750.6 | -27.2 | 0.814 | 20 | 1 | 0 |
|  | 2 | C | 192 | 53 | 11726 | D | 173 | 40 | 11721 | 1747.2 | -27.1 | 0.820 | 20 | 1 | 0 |
|  | 3 | A | 190 | 53 | 11709 | B | 172 | 40 | 11727 | 1744.6 | -27.1 | 0.815 | 20 | 1 | 0 |
|  | 4 | E | 188 | 52 | 11709 | F | 173 | 40 | 11702 | 1741.6 | -27.0 | 0.813 | 21 | 1 | 0 |
|  | 5 | D | 188 | 52 | 11721 | E | 173 | 40 | 11709 | 1740.4 | -27.0 | 0.815 | 20 | 1 | 0 |
|  | 6 | B | 186 | 53 | 11727 | C | 174 | 40 | 11726 | 1740.0 | -27.0 | 0.816 | 20 | 1 | 0 |
|  | Average: |  |  |  |  |  |  |  |  | 1744.1 | -27.1 | 0.816 | 20 | 1 | 0 |
| 2 | 7 | A | 81 | 24 | 11709 | E | 79 | 23 | 11709 | 767.5 | -15.0 | 0.558 | 4 | 1 | 0 |
|  | 8 | D | 81 | 22 | 11721 | F | 81 | 24 | 11702 | 765.2 | -14.8 | 0.582 | 4 | 1 | 0 |
|  | 9 | C | 82 | 22 | 11726 | E | 81 | 24 | 11709 | 761.7 | -14.7 | 0.593 | 4 | 1 | 0 |
|  | 10 | B | 80 | 23 | 11727 | F | 79 | 22 | 11702 | 757.0 | -14.5 | 0.581 | 4 | 1 | 0 |
|  | 11 | B | 81 | 22 | 11727 | D | 80 | 24 | 11721 | 754.1 | -14.7 | 0.587 | 4 | 1 | 0 |
|  | 12 | A | 77 | 23 | 11709 | C | 81 | 24 | 11726 | 749.3 | -14.5 | 0.575 | 4 | 1 | 0 |
|  | Average: |  |  |  |  |  |  |  |  | 759.1 | -14.7 | 0.579 | 4 | 1 | 0 |
| **MAM-∆LP** | | **Structure 1** | | | | **Structure 2** | | | |  |  |  |  |  |  |
| **Id** | **NN** | **Range** | **iNat** | **iNres** | **Surface Å2** | **Range** | **iNat** | **iNres** | **Surface Å2** | **Interface area, Å2** | **ΔiG**  **kcal/mol** | **ΔiG**  **P-value** | **N_HB_** | **N_SB_** | **N_DS_** |
| 1 | 1 | A | 171 | 40 | 9773 | F | 190 | 51 | 9773 | 1701.4 | -26.3 | 0.687 | 17 | 0 | 0 |
|  | 2 | D | 188 | 51 | 9776 | E | 169 | 40 | 9767 | 1697.9 | -26.3 | 0.675 | 17 | 0 | 0 |
|  | 3 | C | 189 | 51 | 9782 | D | 169 | 40 | 9776 | 1695.0 | -26.1 | 0.686 | 17 | 0 | 0 |
|  | 4 | B | 189 | 51 | 9770 | C | 169 | 40 | 9782 | 1694.1 | -26.1 | 0.687 | 17 | 0 | 0 |
|  | 5 | E | 188 | 51 | 9767 | F | 170 | 40 | 9773 | 1693.2 | -26.0 | 0.685 | 17 | 0 | 0 |
|  | 6 | A | 188 | 51 | 9773 | B | 171 | 40 | 9770 | 1691.3 | -26.1 | 0.694 | 16 | 0 | 0 |
|  | Average: |  |  |  |  |  |  |  |  | 1695.5 | -26.2 | 0.686 | 17 | 0 | 0 |
| 2 | 7 | A | 45 | 13 | 9773 | C | 55 | 21 | 9782 | 530.5 | -12.3 | 0.221 | 3 | 1 | 0 |
|  | 8 | C | 45 | 13 | 9782 | E | 53 | 20 | 9767 | 529.9 | -12.3 | 0.210 | 3 | 1 | 0 |
|  | 9 | B | 51 | 21 | 9770 | F | 48 | 13 | 9773 | 528.4 | -12.2 | 0.231 | 3 | 1 | 0 |
|  | 10 | B | 44 | 13 | 9770 | D | 55 | 21 | 9776 | 528.3 | -12.2 | 0.220 | 3 | 1 | 0 |
|  | 11 | A | 53 | 20 | 9773 | E | 43 | 13 | 9767 | 525.7 | -12.2 | 0.198 | 3 | 1 | 0 |
|  | 12 | D | 47 | 13 | 9776 | F | 51 | 20 | 9773 | 523.6 | -12.2 | 0.226 | 3 | 1 | 0 |
|  | Average: |  |  |  |  |  |  |  |  | 527.7 | -12.2 | 0.218 | 3 | 1 | 0 |

**Supplementary table 5**: Protein identification of the excised, most intense band from the lithium chloride extract.

| **Uniprot ID** | **Description** | **log(E-value)** | **Coverage** | **MW (Da)** | **Size (aa)** | **Specific spectra** | **emPAI** |
| --- | --- | --- | --- | --- | --- | --- | --- |
| C7H4X2 | MAM | -149.4513482 | 70.37% | 14,491.36 | 135 | 81 | 4.64 × 10⁹ |

***
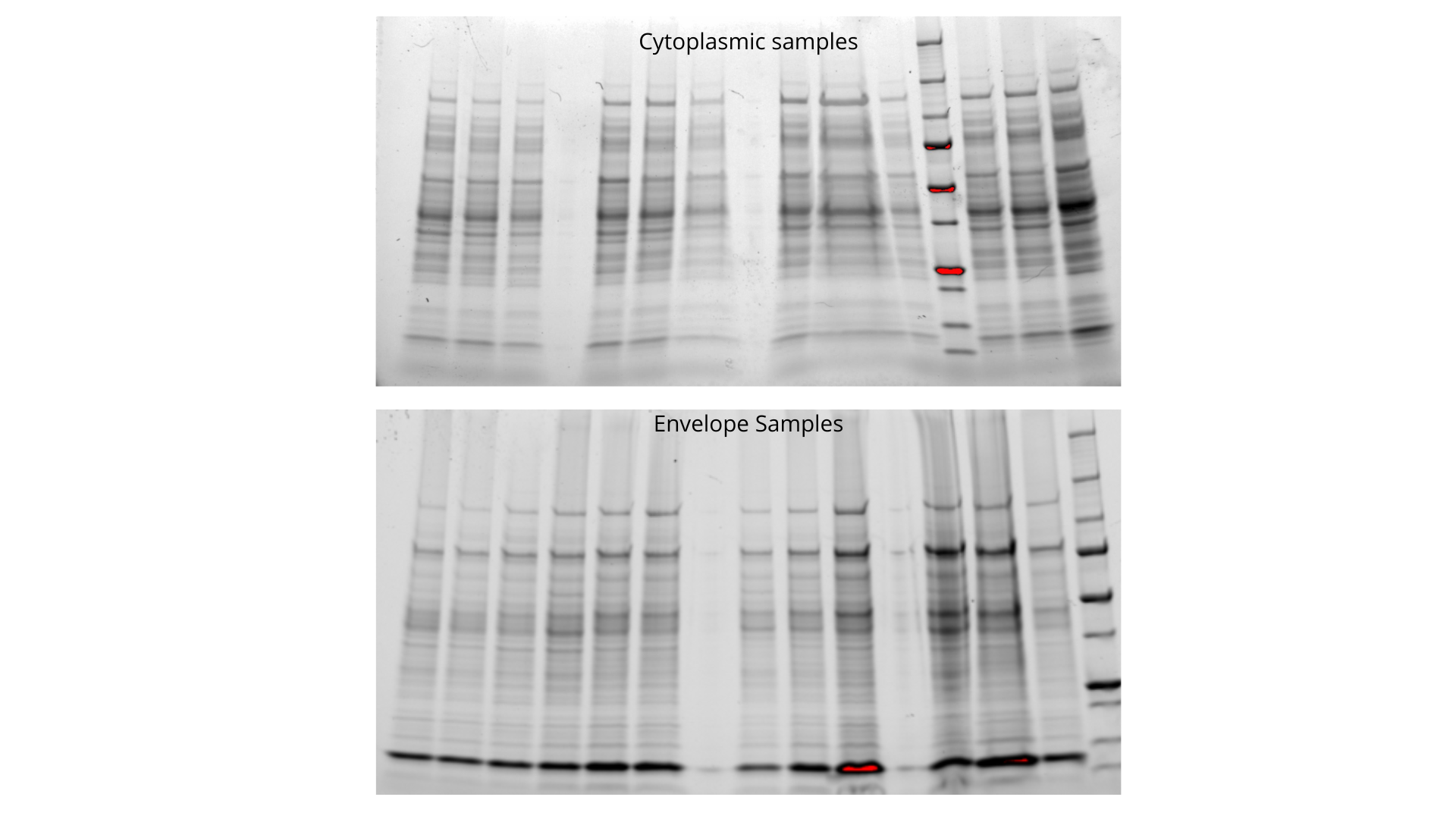
*Supplementary figure 1:** Complete figure of SDS-PAGE gel of cytoplasmic and envelope protein fractions of *F. duncaniae****.***

Complete images SDS-PAGE analysis of cytoplasmic (top) and envelope (bottom) protein extracts from *F. duncaniae*. Approximately 10 µg of protein was loaded into each well. The envelope samples show a prominent band between 10-15 kDa, with a low-intensity band in the corresponding position on the cytoplasmic fraction. Such position is consistent with the size of MAM (14.5 kDa), identifying MAM as an abundant envelope protein.


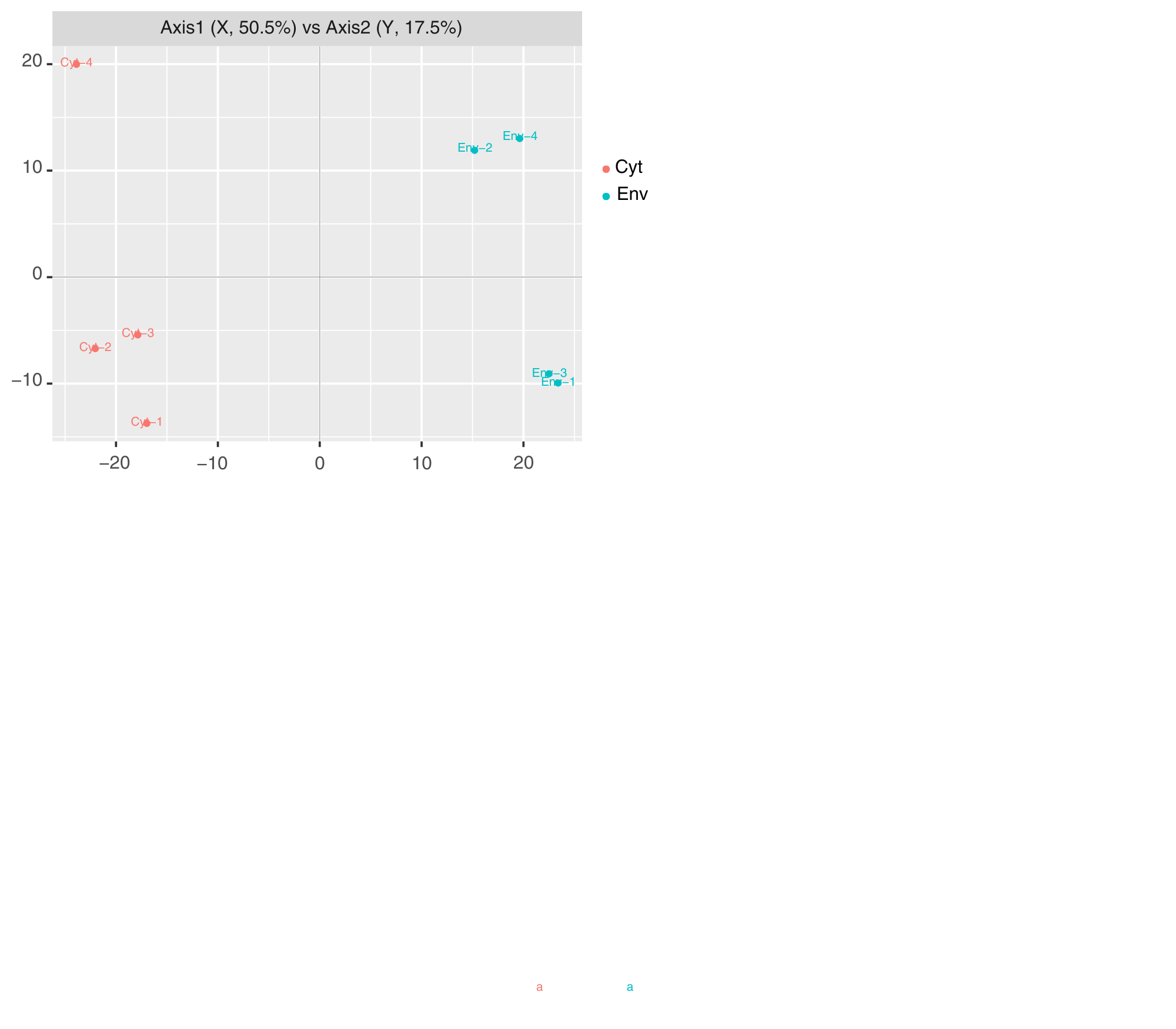
**Supplementary figure 2.** Principal Component Analysis (PCA) of cytoplasmic and envelope samples from *F. duncaniae* culture.

The figure displays the PCA results for cytoplasmic (Cyt - orange) and envelope (Env -blue) samples from *F. duncaniae* cultures. The plots show the separation between the samples across different principal components.


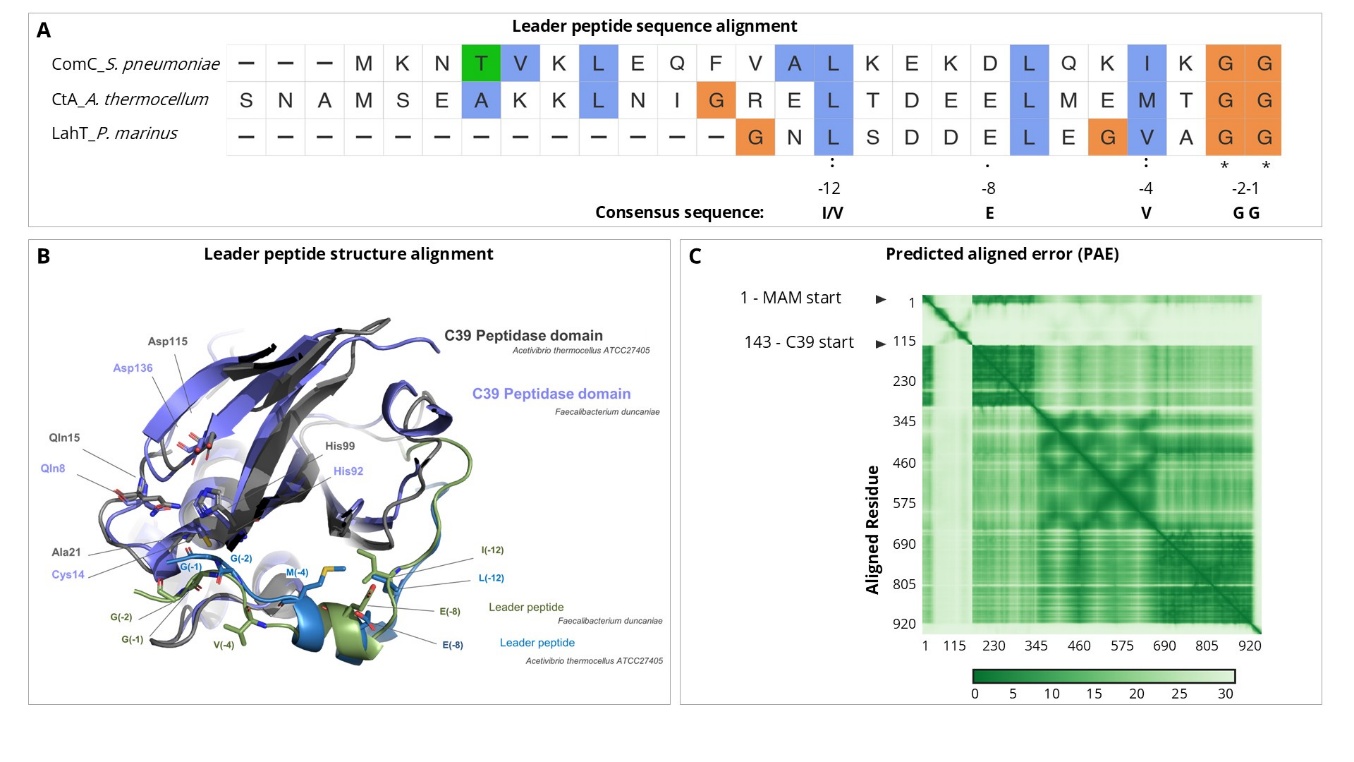
**Supplementary figure 3:** Leader peptide alignment and Predicted Aligned Error (PAE) plot of the interaction PCAT-MAM.

A) Alignment of MAM’s leader peptide with leader peptides from other bacterial species (*S. pneumoniae, C. thermocellum,* and *P. marinus*). The alignment reveals a conserved sequence pattern, with the notable presence of the double glycine motif (GG) at the C-terminal region. Such sequence pattern is crucial for proteolytic cleavage by PCAT. B) Structural alignment of the leader peptide and the C39 peptidase domain of F*. duncaniae* and *A. thermocellus.* C) AlphaFold PAE graph for the MAM-PCAT interaction, illustrating the high accuracy of the N-terminus positioning of MAM relative to the C39 peptidase domain despite lower confidence in the core helices and other PCAT transporter domains. The scale indicates lower expected errors (dark green), while higher expected errors are shown in light green.


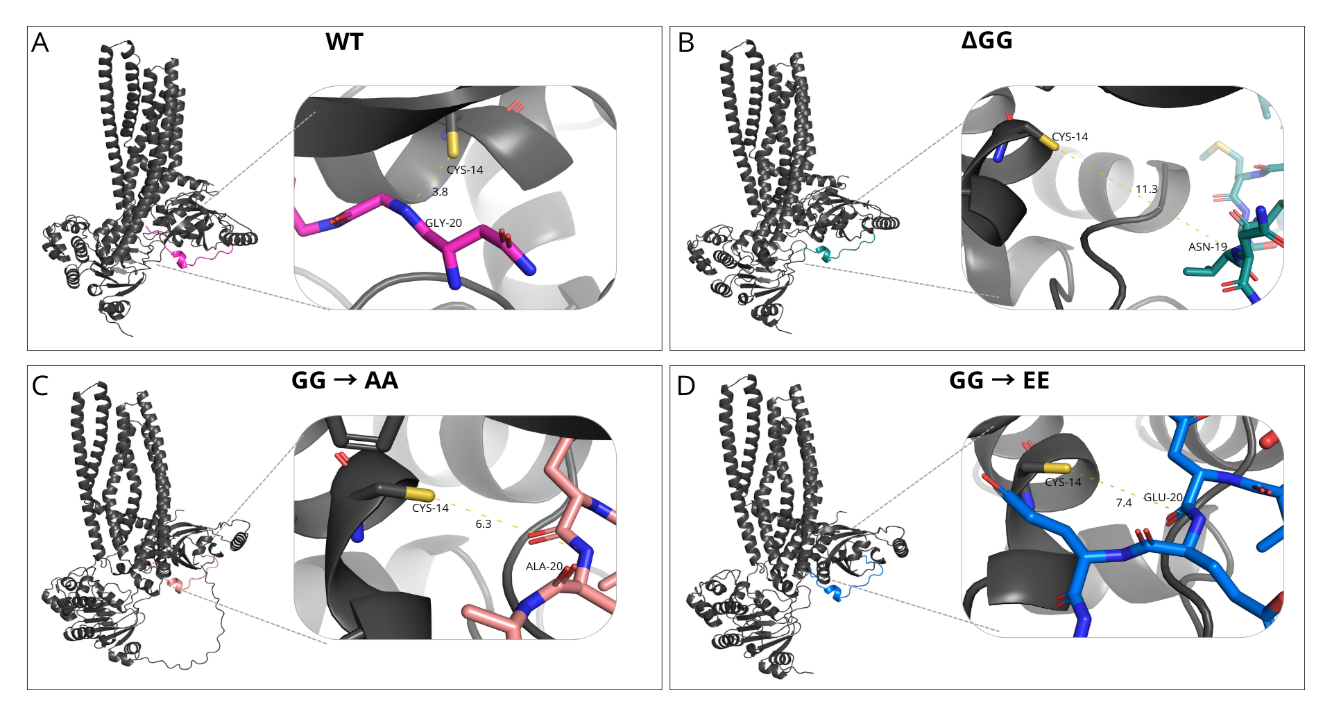
**Supplementary figure 4.** Structural impact of the leader peptide G20G20 motif mutations in the interaction PCAT-MAM**.**

Comparison of wild-type docking with PCAT-ABC (A) and mutant forms: (B) ΔGG, (C) GG→AA, and (D) GG→EE. The peptidase amino acid residue CYS-14 and the respective ligand are indicated in each panel. Yellow dashed lines represent the distance between residues, measured in Å.

**
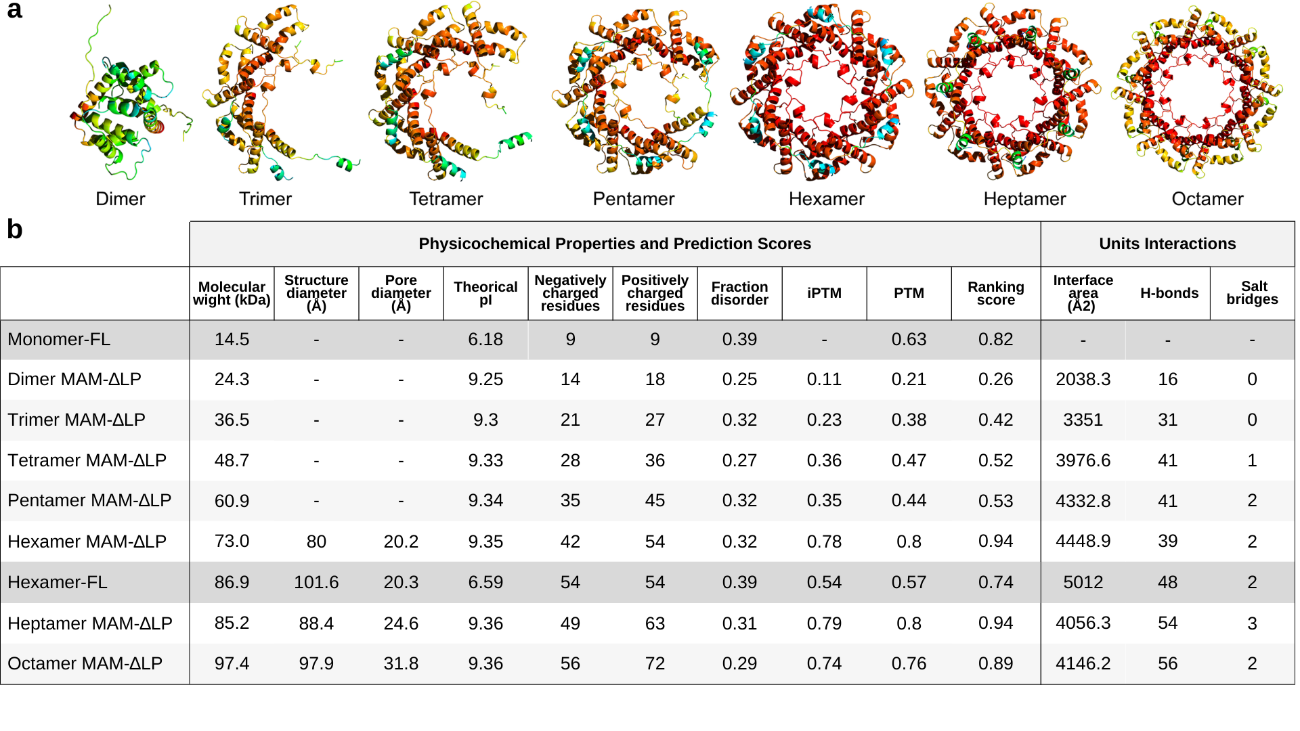
**

**Supplementary Figure 5**: Predicted models of MAM complexes and structure assessment.

A) The figure illustrates the predicted structural models of MAM-∆LP in various oligomeric states (dimer, trimer, tetramer, pentamer, hexamer, heptamer, and octamer) based on AlphaFold3 predictions. The color scale represents plDDT scores, with red indicating high confidence in the structural prediction and blue/green indicating lower confidence. The central pore formation is observed starting from the hexamer, which is hypothesized to be the most stable and significant organization. B) The table presents the physicochemical properties, including molecular weight, number of amino acids, theoretical isoelectric point (pI), and the number of charged residues (negative and positive). It also displays confidence scores from AlphaFold3, including fraction disorder, interface predicted template modeling (iPTM) score, predicted template modeling (pTM) score, and ranking scores. In addition, the right panel of the table shows the sum of the interactions that exist for the MAM unit with the most interactions in the structure, including the surface area contact as well the H-bonds and salt bridges. The hexameric and heptameric predicted structures of MAM-∆LP have equivalent qualitative metrics, although the hexamer is the shape that maximizes the contact area between the monomers.


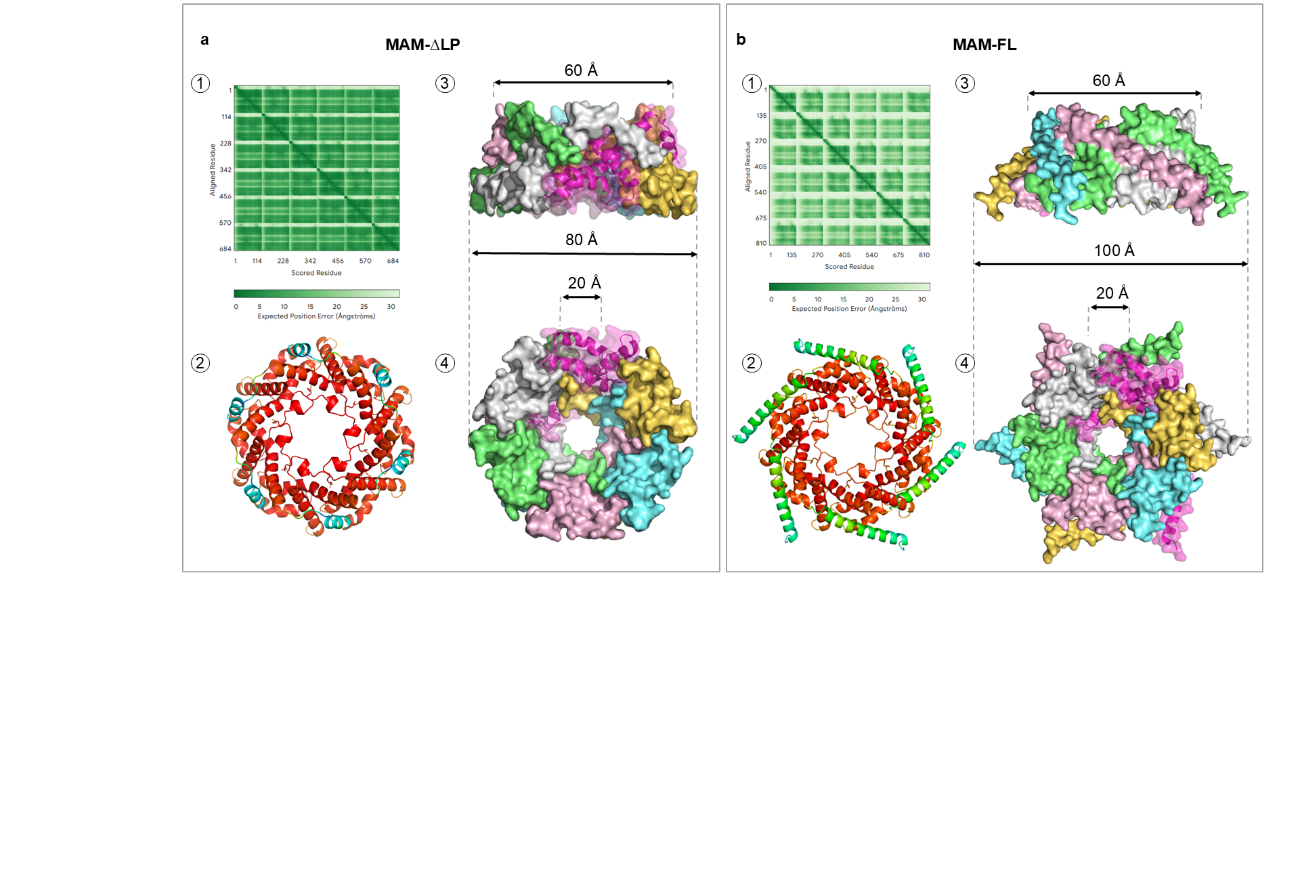
**Supplementary figure 6. Comparison between hexameric models of MAM-ΔLP and MAM-FL.**
A) MAM-ΔLP and B) MAM-FL hexameric structures. Panel 1 shows the Predicted Aligned Error (PAE) plots, indicating model confidence across residue pairs. Panel 2 displays side views of the predicted hexameric structures, colored by pLDDT scores (red: high confidence; green/blue: lower confidence). Panels 3 and 4 show two orientations of the hexamers, highlighting their overall architecture and dimensions. Each monomer is represented in a distinct color to illustrate the hexameric arrangement.

**
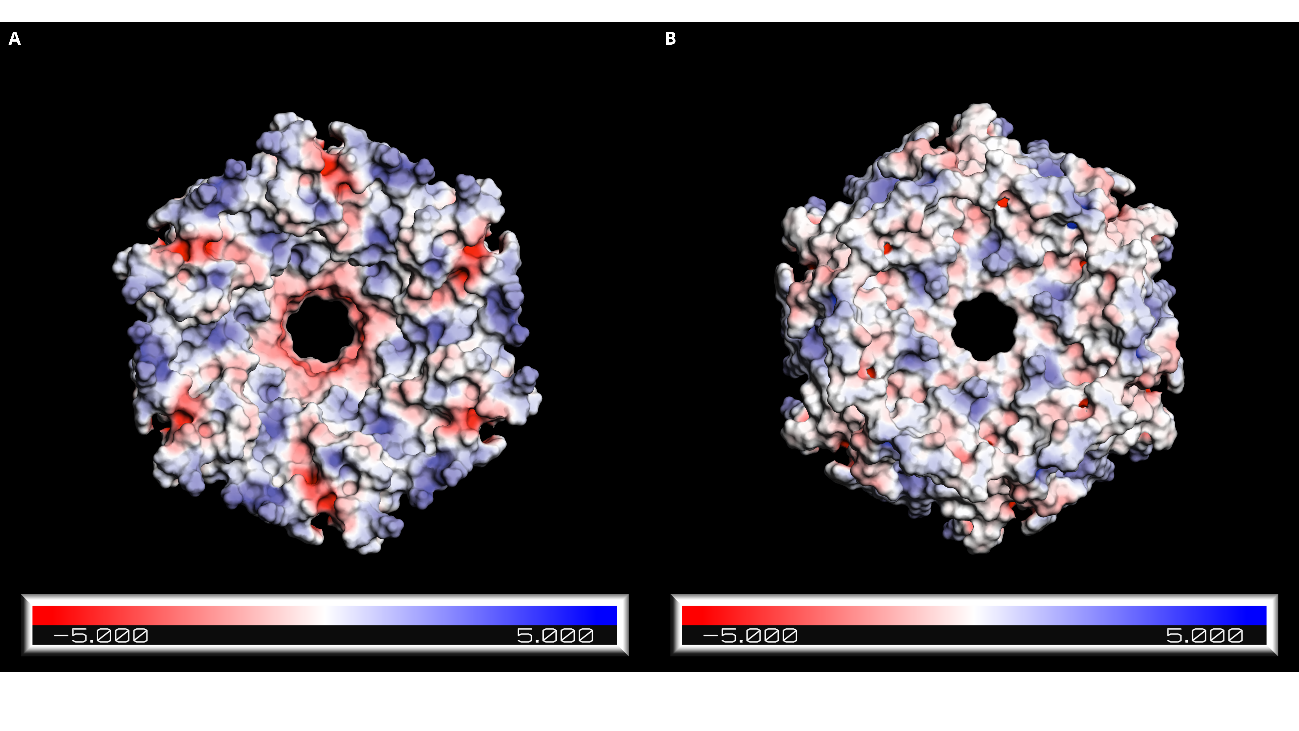
Supplementary figure 7:** Electrostatic potential map of the MAM-∆LP hexameric complex.

The figure illustrates the two faces of the MAM-ΔLP hexameric model, predicted using AlphaFold (A and B). The color scale represents the electrostatic potential, where red regions correspond to negatively charged areas, blue regions represent positively charged areas, and white indicates neutral potential.


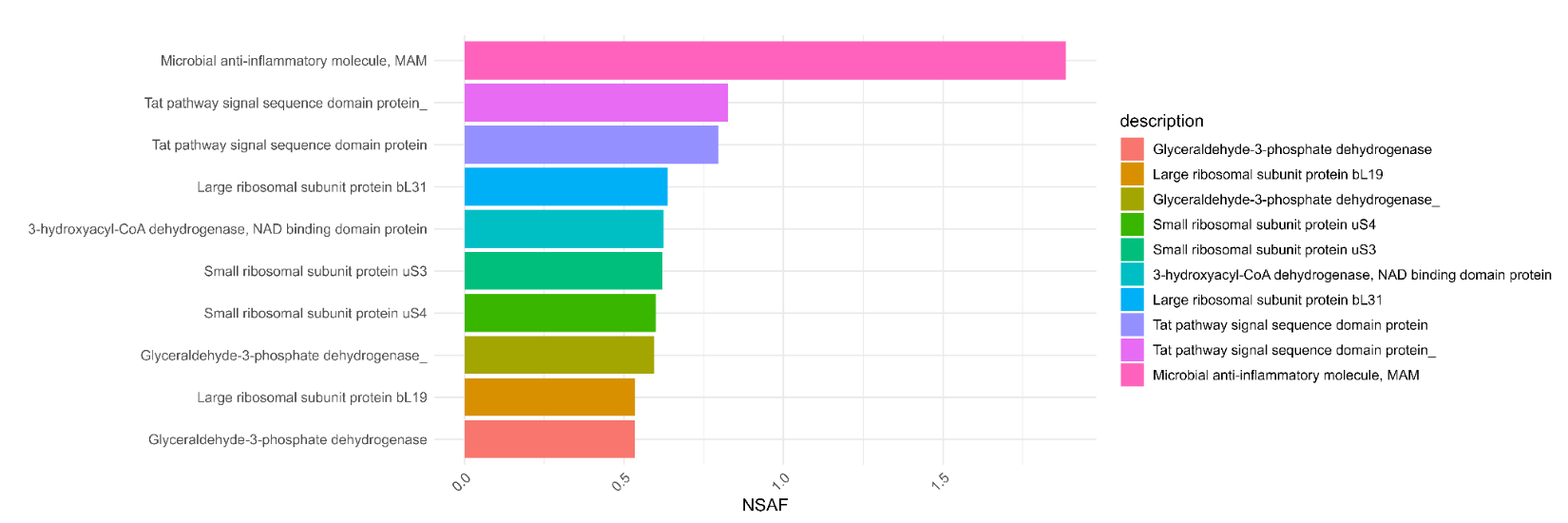
**Supplementary figure 8:** Top ten most abundant protein on the LiCl extraction based on NSAF score.

The plot X-axis indicates the Normalized Spectral Abundance Factor (NSAF) scores of the ten most abundant identified proteins from the LiCl extract. MAM is identified as the most abundant protein of the fraction, with massive abundance compared with the other proteins.


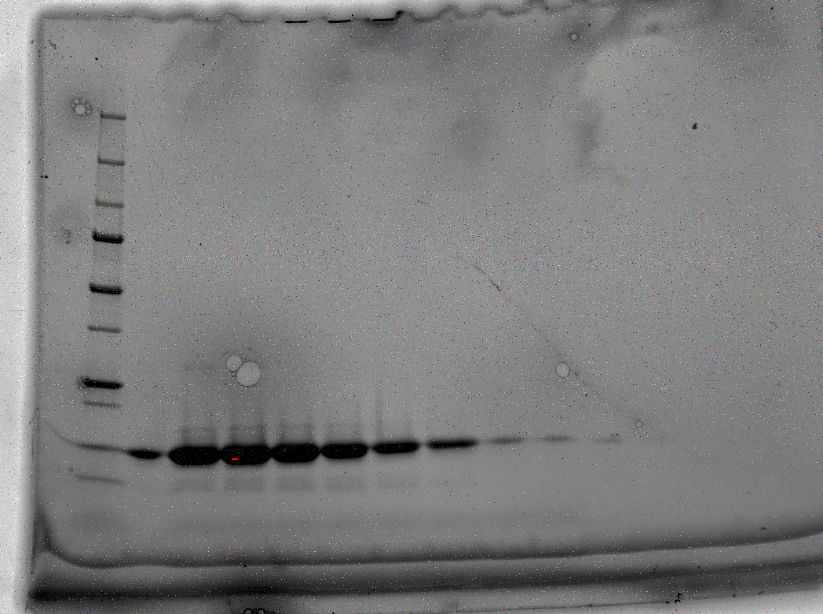
**Supplementary figure 9:** Recombinant MAM purification

The figure shows the electrophoresis gel of the purified recombinant MAM in several concentrations. The bands are higher


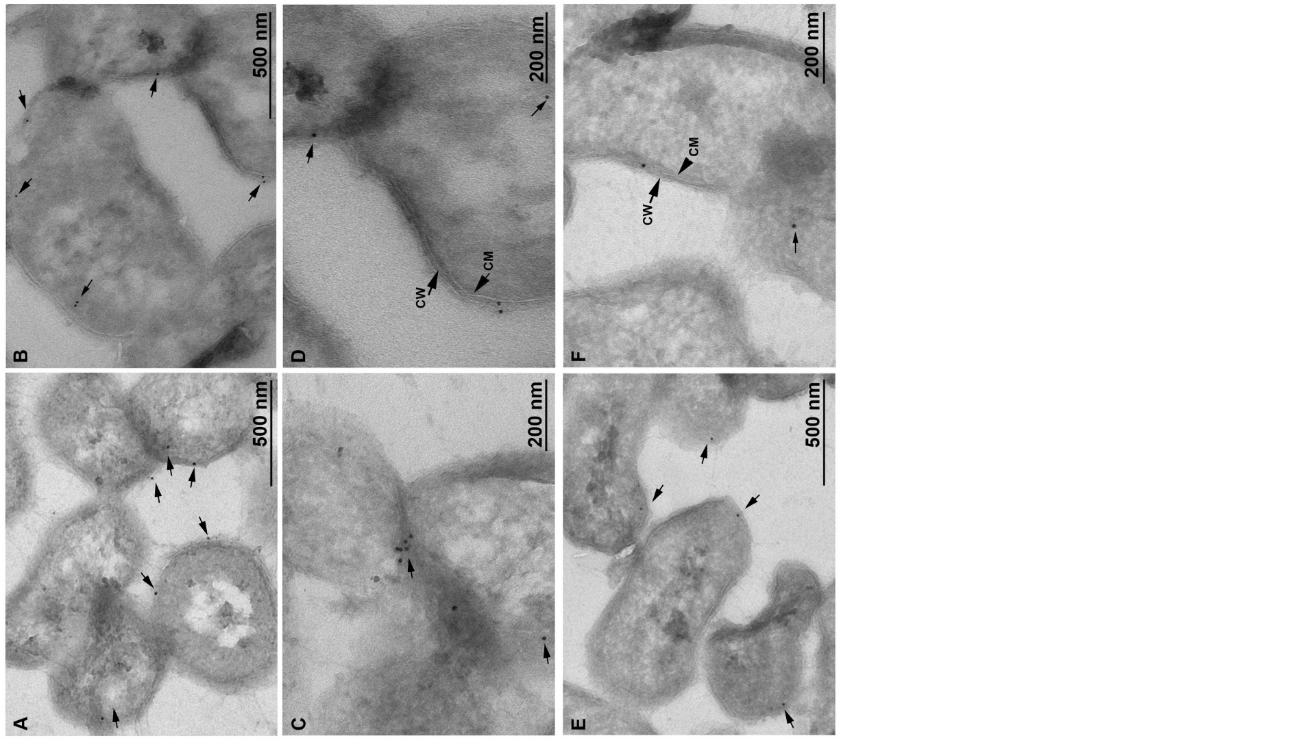
**Supplementary figure 10:** Immunogold labeling of *F. duncaniae* cells.

The figure shows *F. duncaniae* cells labeled with antibodies recognizing R-MAM, indicated by black dots. Arrows point to the locations of the recombinant MAM labeling within the cells.

**Supplementary data 1:** Identification and quantification of peptides in the bacterial supernatant.

Sheet 1 summarizes sample information, including groups, subgroups, protein and peptide counts, PSMs (Peptide-Spectrum Match) and FDR (False Discovery Rate) values. Sheet 2 details peptide identification, including group and subgroup IDs, sequences, modifications, charge, theoretical mass, and abundances across samples.

**Supplementary data 2:** Proteomic identification of Cytoplasm and Envelope fractions.

This dataset includes specific spectra counts for all identified proteins in cytoplasmic and envelope fractions (specific spectra sheet), detailed information on detected peptides (peptidomic compar spectra sheet), and relative protein quantification using NSAF (Normalized Spectral Abundance Factor).

**Supplementary data 3:** LiCl protein identification

The file contains the spectra count and NSAF score for all proteins identified in LiCl extract, evidencing MAM (C7H4X2) as the most abundant protein.
